# Supplementary material for: Mendelian imputation of parental genotypes improves estimates of direct genetic effects
Source: Nat Genet. 2022 Jun 9;54(6):897–905. doi: 10.1038/s41588-022-01085-0 (PMC9197765; doi:10.1038/s41588-022-01085-0)
Supplement: Supplementary file 1 — Supplementary note (PDF). Supplementary tables are provided as separate file (sib_imputation_supplementary_tables_March_18_22). [file 41588_2022_1085_MOESM1_ESM.pdf]

nature portfolio

https://doi.org/10.1038/s41588-022-01085-0

Supplementary information

Mendelian imputation of parental genotypes improves estimates of direct genetic effects

In the format provided by the authors and unedited

Supplementary Note for: ‘Mendelian imputation of parental genotypes improves estimates of direct genetic effects’

May 18, 2022

Contents

1 Models **4**

2 Imputation regression theory **5**

2.1 Consistency of estimates . . . 6
2.2 Unbiasedness of estimates . . . 7
2.3 Unbiasedness of empirical sampling variance-covariance matrix . . . 7

3 Imputation from siblings **9**

3.1 Imputation without IBD . . . 10
3.1.1 Variance of imputed parental genotype . . . 10
3.1.2 Handling IBD errors . . . 11
3.2 Imputation with IBD . . . 11
3.2.1 Multiple siblings . . . 12
3.2.2 Variance of imputed parental genotype . . . 12

4 Estimating effects from siblings **14**

4.1 Without imputation . . . 14
4.2 With imputation . . . 15
4.3 Asymmetrical sibling pairs . . . 16

5 One parent missing **18**

5.1 Imputation without phased data . . . 18
5.1.1 Multiple siblings . . . 19
5.2 Imputation with phased data . . . 19
5.3 Association analysis . . . 20

6 Multivariate Meta-analysis **22**

7 Effect of population structure **23**

7.1 Imputation from siblings . . . 25
7.2 Fixing the bias . . . 27
7.2.1 IBD=0 . . . 27
7.2.2 IBD=1 . . . 29
7.2.3 Combining IBD=1 and IBD=0 . . . 30
7.3 Imputation from parent-offspring pairs . . . 31

8 PGI analysis with assortative mating **32**

8.1 Joint distribution of observed and imputed PGIs . . . 33
8.2 Estimating the direct effect of a PGI . . . 33
8.3 Adjusting for the bias introduced by assortative mating . . . 34

9 Inferring IBD between siblings **34**
9.1 With genotyping errors 36
9.2 Smoothing 37
9.3 Parameter optimization 37

10 Mixed model inference **37**
10.1 Loss function and gradients 38
10.2 Optimisation 39

11 Estimating genome-wide correlations between effects **40**

12 Simulations **41**
12.1 Artificial populations 41
12.2 UK Biobank simulations 41
12.2.1 Simulating assortative mating 42
12.2.2 Simulating vertical transmission 43
12.2.3 Simulating population stratification 43

A Equilibrium distribution of observed and imputed PGIs **45**
A.1 Equilibrium variance of PGI 45
A.2 Equilibrium variance of parental PGI 45
A.3 Equilibrium covariance between offspring and parental PGI 46
A.4 Equilibrium variance of imputed parental PGI 46
A.5 Equilibrium covariance between offspring and imputed parental PGI 48
A.6 Equilibrium covariance between imputed and observed parental PGI 48

B Imputation from one parent and multiple offspring **49**
B.1 With IBD = 0 49
B.2 With IBD = 1 50
B.3 With IBD = 2 52

C Imputation without IBD **54**

D Linear Imputation **55**
D.1 Imputation from parent-offspring pairs 55
D.2 Imputation from sibling pairs 55

1 Models

The primary model whose parameters we aim to estimate is:

$$Y_{ij} = \delta g_{ij} + \alpha_p g_{p(i)} + \alpha_m g_{m(i)} + \epsilon_{ij}. \tag{1}$$

See Supplementary Note Table 1 for definitions of terms. Because offspring genotype varies randomly around parental genotype due to Mendelian segregations during meiosis, δ captures the causal effect of inheriting the allele being counted, along with partial capture of causal effects of other alleles that are linkage disequilibrium with the allele being counted due to physical linkage[1]. The paternal and maternal non-transmitted coefficients (NTCs), α_p and α_m, capture indirect genetic effects from relatives, and the effects of other genetic and environmental factors the allele is correlated with due to non-random mating (population structure and assortative mating). The residual ϵ_{ij} captures all other factors influencing the trait that are not correlated with siblings' or parents' genotypes. We allow for this to be correlated between siblings in the same family: $\text{Corr}(\epsilon_{i1}, \epsilon_{i2}) = r$.

term	definition
g_{ij}	genotype of sibling j in family i
g_{ij}^p	0/1 genotype of paternally inherited allele of sibling j in family i
g_{ij}^m	0/1 genotype of maternally inherited allele of sibling j in family i
$g_{p(i)}$	genotype of father in family i
$g_{m(i)}$	genotype of mother in family i
$g_{\text{par}(i)}$	$g_{\text{par}(i)} = g_{p(i)} + g_{m(i)}$
f	allele frequency: $\mathbb{E}[g_{ij}] = 2f$
Y_{ij}	phenotype of sibling j in family i
δ	direct effect
η_s	indirect genetic effect from sibling
α_p	paternal non-transmitted coefficient
α_m	maternal non-transmitted coefficient
α	average non-transmitted coefficient: $\alpha = (\alpha_p + \alpha_m)/2$
β	population effect: $\beta = \delta + \alpha$
ϵ_{ij}	residual component of Y_{ij} that is uncorrelated with siblings' and parents' genotypes
r	correlation of siblings' residuals: $r = \text{Corr}(\epsilon_{i1}, \epsilon_{i2})$
σ_ϵ^2	variance of siblings' residuals
PGI_{ij}	polygenic index of sibling j in family i
$\text{PGI}_{p(i)}$	polygenic index of the father in family i
$\text{PGI}_{m(i)}$	polygenic index of the mother in family i
$\text{PGI}_{\text{par}(i)}$	$\text{PGI}_{\text{par}(i)} = \text{PGI}_{p(i)} + \text{PGI}_{m(i)}$
r_{am}	$r_{am} = \text{Corr}(\text{PGI}_{p(i)}, \text{PGI}_{m(i)})$

Supplementary Note Table 1: Table of terms.

We also consider a model with indirect effects from siblings:

$$Y_{i1} = \delta g_{i1} + \eta_s g_{i2} + (\alpha_p - \eta_s/2) g_{p(i)} + (\alpha_m - \eta_s/2) g_{m(i)} + \epsilon_{i1}; \tag{2}$$
$$Y_{i2} = \delta g_{i2} + \eta_s g_{i1} + (\alpha_p - \eta_s/2) g_{p(i)} + (\alpha_m - \eta_s/2) g_{m(i)} + \epsilon_{i2}.$$

Here η_s captures the indirect effect of the allele through the sibling, and partly captures indirect effects of other alleles in linkage disequilibrium due to physical linkage. The coefficients on the parental genotypes differ from those in (1) because each parental allele has a 50% chance of being passed onto the sibling, so that α_p and α_m capture one half of the indirect effect from the sibling (if present) in (1). Because both siblings' genotypes vary randomly given the genotypes of the mother and father, estimates of δ and η_s from fitting this model are unbiased[2].

It is useful to also consider models where we model only the sum of maternal and paternal genotypes. This model can be derived from (1) through the identity:

$$\alpha_p g_{p(i)} + \alpha_m g_{m(i)} = \left(\frac{\alpha_p + \alpha_m}{2}\right)(g_{p(i)} + g_{m(i)}) + \left(\frac{\alpha_p - \alpha_m}{2}\right)(g_{p(i)} - g_{m(i)}). \tag{3}$$

This allows us to write (1) as

$$Y_{ij} = \delta g_{ij} + \left(\frac{\alpha_p + \alpha_m}{2}\right)(g_{p(i)} + g_{m(i)}) + \left(\frac{\alpha_p - \alpha_m}{2}\right)(g_{p(i)} - g_{m(i)}) + \epsilon_{ij}. \tag{4}$$

Notice that $g_{p(i)} - g_{m(i)}$ is uncorrelated with g_{ij} and $g_{p(i)} + g_{m(i)}$, so we can subsume the difference term into the residual and write the model as

$$Y_{ij} = \delta g_{ij} + \alpha g_{\text{par}(i)} + \epsilon'_{ij}, \tag{5}$$

where $\alpha = (\alpha_p + \alpha_m)/2$ is the average NTC, $g_{\text{par}(i)} = g_{p(i)} + g_{m(i)}$ is the sum of maternal and paternal genotypes, and ϵ'_{ij} is uncorrelated with g_{ij} and $g_{\text{par}(i)}$. Note that ϵ'_{ij} in (5) is not the same as ϵ_{ij} in (1) if $\alpha_p \neq \alpha_m$. However, this distinction is practically unimportant when $(\alpha_p - \alpha_m)^2$ is small relative to the phenotypic variance. Because g_{ij} varies randomly around $g_{\text{par}(i)}/2$, estimates of δ from fitting (5) are unbiased[3]. Similarly, we can fit a model with indirect effects from siblings that only considers the sum of maternal and paternal genotypes:

$$Y_{i1} = \delta g_{i1} + \eta_s g_{i2} + (\alpha - \eta_s/2) g_{\text{par}(i)} + \epsilon_{i1}; \tag{6}$$
$$Y_{i2} = \delta g_{i2} + \eta_s g_{i1} + (\alpha - \eta_s/2) g_{\text{par}(i)} + \epsilon_{i2}.$$

2 Imputation regression theory

In real data, genotypes of one or both parents are often missing. We consider imputing the missing parental genotypes as the conditional expectation of the missing genotype(s) given the observed genotypes. For example, if we have genotypes on two siblings, we can impute the sum of maternal and paternal genotypes as $\hat{g}_{\text{par}(i)} = \mathbb{E}[g_{\text{par}(i)} | g_{i1}, g_{i2}]$. We first prove that regression using imputation of this kind produces unbiased and consistent estimates of parameters along with unbiased sampling error estimates before considering specific applications.

Lemma 1. Consider two random column vectors X_0 and X_1 and a statistic A, which could be, for example, the IBD state of two siblings at a SNP. Let $\hat{X}_1 = \mathbb{E}[X_1|X_0, A]$, then $\mathrm{Cov}(X_1, \hat{X}_1) = \mathrm{Var}(\hat{X}_1)$ and $\mathrm{Cov}(X_0, \hat{X}_1) = \mathrm{Cov}(X_0, X_1)$.

Proof. First note that $\mathbb{E}[\hat{X}_1] = \mathbb{E}[\mathbb{E}[X_1|X_0, A]] = \mathbb{E}[X_1]$ by the Law of Iterated Expectations. We now compute

$$\mathrm{Cov}(X_1, \hat{X}_1) = \mathbb{E}[X_1 \hat{X}_1^T] - \mathbb{E}[X_1]\mathbb{E}[\hat{X}_1]^T \tag{7}$$

$$= \mathbb{E}[\mathbb{E}[X_1 \hat{X}_1^T | X_0, A]] - \mathbb{E}[\hat{X}_1]\mathbb{E}[\hat{X}_1]^T, \tag{8}$$

by the Law of Iterated Expectations, and using the fact that $\mathbb{E}[\hat{X}_1] = \mathbb{E}[X_1]$. Since conditional on X_0 and A, $\hat{X}_1$ is constant, we have that $\mathbb{E}[\mathbb{E}[X_1 \hat{X}_1^T | X_0, A]] = \mathbb{E}[\mathbb{E}[X_1|X_0, A]\hat{X}_1^T] = \mathbb{E}[\hat{X}_1 \hat{X}_1^T]$, and therefore

$$\mathrm{Cov}(X_1, \hat{X}_1) = \mathbb{E}[\hat{X}_1 \hat{X}_1^T] - \mathbb{E}[\hat{X}_1]\mathbb{E}[\hat{X}_1]^T = \mathrm{Var}(\hat{X}_1). \tag{9}$$

We use the Law of Total Covariance to compute $\mathrm{Cov}(X_0, X_1)$:

$$\mathrm{Cov}(X_0, X_1) = \mathbb{E}[\mathrm{Cov}(X_0, X_1|X_0, A)] + \mathrm{Cov}(\mathbb{E}[X_0|X_0, A], \mathbb{E}[X_1|X_0, A]). \tag{10}$$

Since X_0 is a constant given X_0, $\mathrm{Cov}(X_0, X_1|X_0, A) = 0$. Furthermore, $\mathbb{E}[X_0|X_0, A] = X_0$. Therefore,

$$\mathrm{Cov}(X_0, X_1) = \mathrm{Cov}(X_0, \hat{X}_1). \tag{11}$$

□

2.1 Consistency of estimates

Theorem 2. Let $X = [X_0 \; X_1]$; $\hat{X}_1 = \mathbb{E}[X_1|X_0, A]$; $\hat{X} = [X_0 \; \hat{X}_1]$; and $Y = X\theta + \epsilon$, where $\epsilon \perp X$. Then $\hat{\theta} = (\hat{X}^T \hat{X})^{-1} \hat{X}^T Y$ is a consistent estimator of θ provided that $\mathrm{Var}(\hat{X})$ is invertible.

Proof. Provided that $\mathrm{Var}(\hat{X})$ is invertible,

$$\lim_{n \to \infty} \hat{\theta} = \mathrm{Var}(\hat{X})^{-1} \mathrm{Cov}(\hat{X}, Y). \tag{12}$$

Using Lemma 1, we have that $\mathrm{Cov}(\hat{X}, Y) = \mathrm{Cov}(\hat{X}, X\theta) = \mathrm{Var}(\hat{X})\theta$. Therefore,

$$\lim_{n \to \infty} \hat{\theta} = \mathrm{Var}(\hat{X})^{-1} \mathrm{Var}(\hat{X})\theta = \theta. \tag{13}$$

□

Remark. This also shows we can combine different imputations in a single regression while retaining a consistent estimator for θ. Consider the model as above except split into two subsamples, a of n_a individuals, and b of n_b individuals: $Y = [Y_a \; Y_b]^T = [X_a \; X_b]^T \theta + \epsilon$. We

impute X_a as $\hat{X}_a$ and X_b as $\hat{X}_b$, where $\text{Var}(\hat{X}_a) \neq \text{Var}(\hat{X}_b)$, but where we have $\text{Cov}(\hat{X}_a, X_a) = \text{Var}(\hat{X}_a)$ and $\text{Cov}(\hat{X}_b, X_b) = \text{Var}(\hat{X}_b)$ as above. Then we have that

$$\hat{\theta} \to (n_a\text{Var}(\hat{X}_a) + n_b\text{Var}(\hat{X}_b))^{-1}(n_a\text{Cov}(\hat{X}_a, Y_a) + n_b\text{Cov}(\hat{X}_b, Y_b)) \tag{14}$$

$$= (n_a\text{Var}(\hat{X}_a) + n_b\text{Var}(\hat{X}_b))^{-1}(n_a\text{Var}(\hat{X}_a)\theta + n_b\text{Var}(\hat{X}_b)\theta) = \theta, \tag{15}$$

provided that the resulting combined variance-covariance matrix $(n_a\text{Var}(\hat{X}_a) + n_b\text{Var}(\hat{X}_b))$ is invertible. This is especially useful when $\text{Var}(\hat{X}_a)$ or $\text{Var}(\hat{X}_b)$ is not invertible since the imputation in that sample is not of full rank, but the resulting combined regression is of full-rank. See also Section 6 on multivariate meta-analysis.

Corollary 2.1. This implies that the generalised least-squares (GLS) estimator is also consistent since $\text{Var}(\hat{X}) = \text{Cov}(\hat{X}, X)$ implies that $B\text{Var}(\hat{X})B^T = B\text{Cov}(\hat{X}, X)B^T$, which implies $\text{Var}(B\hat{X}) = \text{Cov}(B\hat{X}, BX)$. Let $\text{Cov}(\epsilon) = \Sigma$, then the GLS estimator of θ is

$$\hat{\theta}_{\text{gls}} = (\hat{X}^T\Sigma^{-1}\hat{X})^{-1}\hat{X}^T\Sigma^{-1}Y \to \text{Var}(\Sigma^{-1/2}\hat{X})^{-1}\text{Cov}(\Sigma^{-1/2}\hat{X}, \Sigma^{-1/2}X)\theta = \theta. \tag{16}$$

2.2 Unbiasedness of estimates

We now prove that both the OLS and GLS estimators are unbiased.

Theorem 3. Let $X = [X_0\ X_1]$; $\hat{X}_1 = \mathbb{E}[X_1|X_0, A]$; $\hat{X} = [X_0\ \hat{X}_1]$; and $Y = X\theta + \epsilon$, where $\epsilon \perp X$. Then the GLS estimator $\hat{\theta}_{\text{gls}} = (\hat{X}^T\Sigma^{-1}\hat{X})^{-1}\hat{X}^T\Sigma^{-1}Y$ is an unbiased estimator of θ provided that $(\hat{X}^T\Sigma^{-1}\hat{X})$ is invertible.

$$\mathbb{E}[\hat{\theta}_{\text{gls}}] = \mathbb{E}[(\hat{X}^T\Sigma^{-1}\hat{X})^{-1}\hat{X}^T\Sigma^{-1}Y]; \tag{17}$$

$$= \mathbb{E}[(\hat{X}^T\Sigma^{-1}\hat{X})^{-1}\hat{X}^T\Sigma^{-1}X]\theta; \tag{18}$$

$$= \mathbb{E}[\mathbb{E}[(\hat{X}^T\Sigma^{-1}\hat{X})^{-1}\hat{X}^T\Sigma^{-1}X|X_0, A]]\theta \tag{19}$$

$$= \mathbb{E}[(\hat{X}^T\Sigma^{-1}\hat{X})^{-1}\hat{X}^T\Sigma^{-1}\mathbb{E}[X|X_0, A]]\theta \tag{20}$$

$$= \mathbb{E}[(\hat{X}^T\Sigma^{-1}\hat{X})^{-1}\hat{X}^T\Sigma^{-1}\hat{X}]\theta \tag{21}$$

$$= \theta. \tag{22}$$

Note that this also applies to OLS by setting $\Sigma^{-1} = \text{I}$.

2.3 Unbiasedness of empirical sampling variance-covariance matrix

Here, we demonstrate that the empirical sampling variance-covariance matrix derived from OLS gives an unbiased estimate of the true sampling variance-covariance matrix for OLS estimation from a sample of n independent and identically distributed observations. As in Theorem 2, let $X = [X_0\ X_1]$; $\hat{X}_1 = \mathbb{E}[X_1|X_0, A]$; $\hat{X} = [X_0\ \hat{X}_1]$; and $Y = X\theta + \epsilon = X_0\theta_0 + X_1\theta_1 + \epsilon$, where $\epsilon \perp X$, $\mathbb{E}[\epsilon] = 0$, $\text{Var}(\epsilon) = \sigma^2\text{I}_n$, and $\theta = [\theta_0, \theta_1]^T$ is length p and $\hat{X}^T\hat{X}$ is an invertible $[p \times p]$ matrix.

Lemma 4. The variance of the OLS estimator, $\hat{\theta} = (\hat{X}^T\hat{X})^{-1}\hat{X}^T Y$, is

$$\mathrm{Var}(\hat{\theta}) = \left(\sigma^2 + \theta_1^T[\mathrm{Var}(X_1) - \mathrm{Var}(\hat{X}_1)]\theta_1\right)(\hat{X}^T\hat{X})^{-1} \tag{23}$$

Proof. We can express the phenotype vector Y as $Y = \hat{X}\theta + (X - \hat{X})\theta + \epsilon$, and therefore

$$\hat{\theta} = (\hat{X}^T\hat{X})^{-1}\hat{X}^T(\hat{X}\theta + (X - \hat{X})\theta + \epsilon) = \theta + (\hat{X}^T\hat{X})^{-1}\hat{X}^T[(X_1 - \hat{X}_1)\theta_1 + \epsilon]. \tag{24}$$

From this, we compute

$$\mathrm{Var}(\hat{\theta}) = (\hat{X}^T\hat{X})^{-1}\hat{X}^T\mathbb{E}[(X_1 - \hat{X}_1)\theta_1\theta_1^T(X_1 - \hat{X}_1)^T]\hat{X}(\hat{X}^T\hat{X})^{-1} + \sigma^2(\hat{X}^T\hat{X})^{-1}, \tag{25}$$

where we have used the fact that $\epsilon \perp X$ to remove the covariance terms. Since the samples are independent and identically distributed,

$$\mathbb{E}[(X_1 - \hat{X}_1)\theta_1\theta_1^T(X_1 - \hat{X}_1)^T] = \theta_1^T[\mathrm{Var}(X_1) - \mathrm{Var}(\hat{X}_1)]\theta_1\mathrm{I}_n, \tag{26}$$

where we have used the fact that $\mathrm{Cov}(X_1, \hat{X}_1) = \mathrm{Var}(\hat{X}_1)$ from Lemma 1 to compute the variance of the diagonal elements. Therefore,

$$\mathrm{Var}(\hat{\theta}) = \left(\sigma^2 + \theta_1^T[\mathrm{Var}(X_1) - \mathrm{Var}(\hat{X}_1)]\theta_1\right)(\hat{X}^T\hat{X})^{-1}. \tag{27}$$

□

Theorem 5. Let $\hat{\epsilon} = Y - \hat{X}\hat{\theta}$ be the vector of fitted residuals from OLS regression. We define the empirical sampling variance-covariance matrix to be:

$$\hat{V}_\theta = \frac{\hat{\epsilon}^T\hat{\epsilon}}{n-p}(\hat{X}^T\hat{X})^{-1}. \tag{28}$$

The empirical sampling variance-covariance matrix is an unbiased estimator of the true sampling variance-covariance matrix of $\hat{\theta}$: $\mathbb{E}[\hat{V}_\theta] = \mathrm{Var}(\hat{\theta})$.

Proof. We compute the expectation of the empirical sampling variance-covariance matrix:

$$\mathbb{E}[\hat{V}_\theta] = \frac{\mathbb{E}[\hat{\epsilon}^T\hat{\epsilon}]}{n-p}(\hat{X}^T\hat{X})^{-1}. \tag{29}$$

To compute $\mathbb{E}[\hat{\epsilon}^T\hat{\epsilon}]$ we define the 'hat-matrix',

$$H = \hat{X}(\hat{X}^T\hat{X})^{-1}\hat{X}^T. \tag{30}$$

We can express the residual vector in terms of the hat-matrix:

$$\hat{\epsilon} = (\mathrm{I}_n - H)(X_1 - \hat{X}_1)\theta_1 + (\mathrm{I}_n - H)\epsilon. \tag{31}$$

Therefore,

$$\hat{\epsilon}^T\hat{\epsilon} = \theta_1^T(X_1 - \hat{X}_1)^T(\mathrm{I}_n - H)(X_1 - \hat{X}_1)\theta_1 + \epsilon^T(\mathrm{I}_n - H)\epsilon + \tag{32}$$

$$\theta_1^T(X_1 - \hat{X}_1)^T(\mathrm{I}_n - H)\epsilon^T + \epsilon^T(\mathrm{I}_n - H)(X_1 - \hat{X}_1)\theta_1, \tag{33}$$

where we have used the fact that $(\mathrm{I}_n - H)$ is a projection matrix, so $(\mathrm{I}_n - H)(\mathrm{I}_n - H)^T = (\mathrm{I}_n - H)$. We now use the trace operator to express the first two terms differently:

$$\theta_1^T(X_1 - \hat{X}_1)^T(\mathrm{I}_n - H)(X_1 - \hat{X}_1)\theta_1 = \mathrm{tr}\left((\mathrm{I}_n - H)(X_1 - \hat{X}_1)\theta_1\theta_1^T(X_1 - \hat{X}_1)^T\right); \tag{34}$$

$$\epsilon^T(\mathrm{I}_n - H)\epsilon = \mathrm{tr}\left((\mathrm{I}_n - H)\epsilon\epsilon^T\right). \tag{35}$$

Taking the expectation of $\hat{\epsilon}^T\hat{\epsilon}$, the cross-product terms vanish, giving:

$$\mathbb{E}[\hat{\epsilon}^T\hat{\epsilon}] = \mathrm{tr}\left((\mathrm{I}_n - H)\mathbb{E}[(X_1 - \hat{X}_1)\theta_1\theta_1^T(X_1 - \hat{X}_1)^T]\right) + \mathrm{tr}\left((\mathrm{I}_n - H)\mathbb{E}[\epsilon\epsilon^T]\right). \tag{36}$$

Here, we have used the fact that $\mathbb{E}[\mathrm{tr}(AX)] = \mathrm{tr}(A\mathbb{E}[X])$ when A is a constant matrix. Since $\mathbb{E}[\epsilon\epsilon^T] = \sigma^2\mathrm{I}_n$, and using Equation 26, we obtain

$$\mathbb{E}[\hat{\epsilon}^T\hat{\epsilon}] = (\theta_1^T[\mathrm{Var}(X_1) - \mathrm{Var}(\hat{X}_1)]\theta_1 + \sigma^2)\mathrm{tr}(\mathrm{I}_n - H). \tag{37}$$

All that remains is to compute the trace:

$$\mathrm{tr}(\mathrm{I}_n - H) = \mathrm{tr}(\mathrm{I}_n) - \mathrm{tr}(\hat{X}(\hat{X}^T\hat{X})^{-1}\hat{X}^T) = n - \mathrm{tr}(\mathrm{I}_p) = n - p. \tag{38}$$

Therefore,

$$\mathbb{E}[\hat{V}_\theta] = \left(\sigma^2 + \theta_1^T[\mathrm{Var}(X_1) - \mathrm{Var}(\hat{X}_1)]\theta_1\right)(\hat{X}^T\hat{X})^{-1} = \mathrm{Var}(\hat{\theta}). \tag{39}$$

□

3 Imputation from siblings

Here we outline how to impute parental genotype from siblings without using IBD data, using phased data and IBD, and using un-phased data and IBD. We give the imputed parental genotypes as a function of the genotypes of a sibling pair and, if applicable, the IBD state of the siblings; and we compute the variance of the resulting imputed parental genotypes, which is used in the next section to compute the sampling variance of the estimates.

3.1 Imputation without IBD

For a family of two siblings, the simplest form of imputation imputes the sum of the parents' genotypes as $\hat{g}_{\text{par}(i)} = \mathbb{E}[g_{\text{par}(i)}|g_{i1}, g_{i2}]$. The values are[4] :

		g_{i2}		
		0	1	2
	0	$2f/(2-f)$	$2/(2-f)$	2
g_{i1}	1	$2/(2-f)$	$2+(2f-1)/(1+f(1-f))$	$2(1+2f)/(1+f)$
	2	2	$2(1+2f)/(1+f)$	$2(1+3f)/(1+f)$

Supplementary Note Table 2: $\mathbb{E}[g_{\text{par}(i)}|g_{i1}, g_{i2}]$.

3.1.1 Variance of imputed parental genotype

To compute the variance of the estimator, we need to compute the variance of the imputed parental genotype:

$$\text{Var}(\hat{g}_{\text{par}(i)}) = \sum_{g_{i1},g_{i2}} (\hat{g}_{\text{par}(i)} - 4f)^2 \mathbb{P}(g_{i1}, g_{i2}) \tag{40}$$

The joint distribution of sibling genotypes can be derived by conditioning on the parental genotypes:

$$\mathbb{P}(g_{i1}, g_{i2}) = \sum_{g_{m(i)},g_{p(i)}} \mathbb{P}(g_{i1}, g_{i2}|g_{m(i)}, g_{p(i)})\mathbb{P}(g_{m(i)}, g_{p(i)}). \tag{41}$$

Since sibling genotypes are determined by independent random segregations in the parents, they are conditionally independent given parental genotype. Therefore,

$$\mathbb{P}(g_{i1}, g_{i2}) = \sum_{g_{m(i)},g_{p(i)}} \mathbb{P}(g_{i1}|g_{m(i)}, g_{p(i)})\mathbb{P}(g_{i2}|g_{m(i)}, g_{p(i)})\mathbb{P}(g_{m(i)}, g_{p(i)}). \tag{42}$$

Under assumptions of random mating, the parental genotypes are independent. Therefore,

$$\mathbb{P}(g_{i1}, g_{i2}) = \sum_{g_{m(i)},g_{p(i)}} \mathbb{P}(g_{i1}|g_{m(i)}, g_{p(i)})\mathbb{P}(g_{i2}|g_{m(i)}, g_{p(i)})\mathbb{P}(g_{m(i)})\mathbb{P}(g_{p(i)}). \tag{43}$$

The above probabilities can be computed (laboriously) by application of Mendelian laws of inheritance and using parental genotype frequencies at Hardy-Weinberg equilibrium.

		g_{i2}		
		0	1	2
	0	$(1-f)^2(1-f/2)^2$	$f(1-f)^2(1-f/2)$	$f^2(1-f)^2/4$
g_{i1}	1	$f(1-f)^2(1-f/2)$	$f(1-f)[1+f(1-f)]$	$f^2(1-f)(1+f)/2$
	2	$f^2(1-f)^2/4$	$f^2(1-f)(1+f)/2$	$f^2(1+f)^2/4$

Supplementary Note Table 3: The joint distribution for two siblings' genotypes: $\mathbb{P}(g_{i1}, g_{i2})$.

The variance of the imputed parental genotypes can be computed from the above joint distribution of sibling genotypes and the corresponding values of the above imputed parental genotypes. We do not give an expression here due to its complexity.

3.1.2 Handling IBD errors

We use a generalisation of the above approach to impute the missing parental genotypes when we observe an impossible genotype-IBD state. We make the assumption that if the observed genotypes are impossible given the IBD state, that the IBD state is in error, since errors in IBD inference are typically far more common than errors in genotypes. We therefore impute ignoring the IBD information (Appendix C).

3.2 Imputation with IBD

Let $g_{\text{par}(i)} = g_{m(i)} + g_{p(i)}$ be the sum of the parental genotypes for individual i. This can also be written in terms of the parental alleles: $g_{\text{par}(i)} = g^p_{m(i)} + g^m_{m(i)} + g^p_{p(i)} + g^m_{p(i)}$, where $g^p_{m(i)}$ is the paternally inherited allele of the mother of i, and $g^m_{p(i)}$ is the maternally inherited allele of the father of i. For now, we assume that we know which alleles are shared IBD in addition to the overall IBD state (0, 1, or 2), which, for IBD state 1, requires phased data when both siblings are heterozygous. We construct the estimate of $g_{\text{par}(i)}$, $\hat{g}_{\text{par}(i)}$, to be the expectation of $g_{\text{par}(i)}$ given the observed sibling genotypes and the IBD state of the siblings: $\hat{g}_{\text{par}(i)} = \mathbb{E}[g_{\text{par}(i)}|g_{i1}, g_{i2}, \text{IBD}]$. This gives:

$$\hat{g}_{\text{par}(i)} = \begin{cases} g_{i1} + g_{i2} = g_{\text{par}(i)}, & \text{if IBD} = 0 \\ g_{i1} + g^k_{i2} + f, & \text{if IBD} = 1 \\ g_{i1} + 2f, & \text{if IBD} = 2, \end{cases} \tag{44}$$

where $k \in \{m, p\}$ is such that g^k_{i2} is not IBD with the alleles inherited by sibling 1 in family i.

If we do not have access to phased data, then if both siblings are heterozygous and the IBD state is 1, then the shared allele cannot be determined. In this case, the shared allele is the allele with frequency f with probability $1-f$, and the shared allele is the allele with frequency $1-f$ with probability f. This can be derived from considering the relative frequencies of the three observed parental genotypes: when the allele with frequency f is shared, the probability of observing those three parental alleles is $f(1-f)^2$; and when the allele with frequency $(1-f)$ is observed, the probability of observing those three parental alleles is $f^2(1-f)$. Conditional on both siblings being heterozygous and being in IBD state 1, the probability that the allele with frequency f is shared is $f(1-f)^2/[f(1-f)^2+f^2(1-f)] = f(1-f)^2/f(1-f) = 1-f$; this implies that the probability that the allele with frequency $1-f$ is shared is f. The imputed parental genotype is therefore the average over these two possibilities:

$$\mathbb{E}[g_{\text{par}(i)}|g_{i1} = 1, g_{i2} = 1, \text{IBD} = 1] = f(2+f) + (1-f)(1+f) = 1 + 2f. \tag{45}$$

Let H_i be the event that both siblings are heterozygous, then the imputed parental genotype without phased data is:

$$\hat{g}_{\text{par}(i)} = \begin{cases} g_{i1} + g_{i2} = g_{\text{par}(i)}, & \text{if IBD} = 0 \\ g_{i1} + g_{i2}^{k} + f, & \text{if IBD} = 1 \text{ and } \neg H_i \\ 1 + 2f, & \text{if IBD} = 1 \text{ and } H_i \\ g_{i1} + 2f, & \text{if IBD} = 2, \end{cases} \tag{46}$$

3.2.1 Multiple siblings

First we prove that, for three or more siblings, it is impossible for all pairs of siblings to be in an IBD1 state.

Consider three siblings in family i. We write the genotype of a sibling in terms of parental alleles as $(g^{j}_{m(i)}, g^{k}_{p(i)})$ for $j, k \in \{m, p\}$. Without loss of generality, consider that the genotype of sibling 1 is $(g^{p}_{m(i)}, g^{p}_{p(i)})$ and that the genotype of sibling 2 is $(g^{p}_{m(i)}, g^{m}_{p(i)})$, so that sibling 1 and 2 are in IBD1. We now consider if it is possible for a third sibling to be IBD1 with both sibling 1 and sibling 2.

If sibling 3 inherits the same parental alleles as either sibling 1 or sibling 2, then sibling 3 is IBD2 with another sibling. There are two more possible inheritance patterns for sibling 3: $(g^{m}_{m(i)}, g^{p}_{p(i)})$, which implies sibling 3 is IBD0 with sibling 2; and $(g^{m}_{m(i)}, g^{m}_{p(i)})$, which implies sibling 3 is IBD0 with sibling 1. Therefore, it is impossible for sibling 3 to be IBD1 with both sibling 1 and sibling 2.

This implies that, to impute parental genotypes with more than two siblings, the problem can be reduced to imputing exactly the parental alleles when at least one sibling pair is in an IBD0 state; or imputing as if one has observed a single sibling pair in an IBD2 state if all siblings are in an IBD2 state with each; or reduced to the problem of imputing from a sibling pair in IBD state 1 by reducing sets of siblings that are all IBD 2 with each other to a single sibling. (An additional sibling that is in an IBD2 state with an existing sibling adds no further observed parental alleles.)

3.2.2 Variance of imputed parental genotype

We compute the variance of the parental genotype imputed from a sibling pair. The variance of the imputed parental genotype can be computed by the Law of Total Variance:

$$\text{Var}(\hat{g}_{\text{par}(i)}) = \mathbb{E}_{\text{IBD}}[\text{Var}(\hat{g}_{\text{par}(i)}|\text{IBD})] + \text{Var}_{\text{IBD}}(\mathbb{E}[\hat{g}_{\text{par}(i)}|\text{IBD}]) = \mathbb{E}_{\text{IBD}}[\text{Var}(\hat{g}_{\text{par}(i)}|\text{IBD})], \tag{47}$$

since the expectation of the imputed parental genotypes does not depend upon the IBD state of the siblings. When using phased data for imputation, the variance of the imputed parental

genotype is directly proportional to the number of observed parental alleles:

$$\mathrm{Var}(\hat{g}_{\mathrm{par}(i)}|\mathrm{IBD}) = \begin{cases} 4f(1-f), & \text{if IBD} = 0 \\ 3f(1-f), & \text{if IBD} = 1 \\ 2f(1-f), & \text{if IBD} = 2 \end{cases} . \tag{48}$$

Therefore, since $\mathbb{P}(\mathrm{IBD} = 0) = 0.25$, $\mathbb{P}(\mathrm{IBD} = 1) = 0.5$, and $\mathbb{P}(\mathrm{IBD} = 2) = 0.25$, $\mathrm{Var}(\hat{g}_{\mathrm{par}(i)}) = 3f(1-f) = (3/4)\mathrm{Var}(g_{\mathrm{par}(i)})$. The imputed parental genotype thus captures three quarters of the variance of the observed parental genotype.

Without phased data, the variation in the parental genotype captured by the imputation is decreased due to the inability to determine the shared allele when both siblings are heterozygous and in IBD state 1. To compute the variance of the imputed parental genotype, we need to compute the variance of the imputed parental genotype given IBD state 1. The imputed parental genotype as a function of the observed sibling genotypes given IBD state 1 is:

		g_{i2}		
		0	1	2
	0	f	$1+f$	-
g_{i1}	1	$1+f$	$1+2f$	2+f
	2	-	$2+f$	$3+f$

Supplementary Note Table 4: $\mathbb{E}[g_{\mathrm{par(i)}}|g_{i1}, g_{i2}, \mathrm{IBD} = 1]$

By considering the probability of observing the three observed parental alleles, one can derive the distribution of the observed sibling genotypes given that the IBD state is 1:

		g_{i2}		
		0	1	2
	0	$(1-f)^3$	$f(1-f)^2$	0
g_{i1}	1	$f(1-f)^2$	$f(1-f)$	$f^2(1-f)$
	2	0	$f^2(1-f)$	f^3.

Supplementary Note Table 5: $\mathbb{P}(g_{i1}, g_{i2}|\mathrm{IBD} = 1)$

From these two tables, one can compute that

$$\mathrm{Var}(\hat{g}_{\mathrm{par(i)}}|\mathrm{IBD} = 1) = [3 - f(1-f)]f(1-f); \tag{49}$$

and therefore

$$\mathrm{Var}(\hat{g}_{\mathrm{par(i)}}) = [3 - f(1-f)/2]f(1-f). \tag{50}$$

This shows that the fraction of the variance in the parental genotype captured by imputation without phased data is:

$$\frac{\text{Var}(\hat{g}_{\text{par(i)}})}{\text{Var}(g_{\text{par(i)}})} = \frac{3 - f(1-f)/2}{4}, \tag{51}$$

which decreases with increasing heterozygosity.

4 Estimating effects from siblings

For the analyses in this section, we first assume that $\eta_s = 0$. We then relax the assumption that $\eta_s = 0$ and we consider also the case where sibling pairs are asymmetrical, which may be the case when they differ in sex or age etc.

The model for the two siblings' phenotypes is:

$$Y_{i1} = \delta g_{i1} + \alpha g_{\text{par(i)}} + \epsilon_{i1}; \tag{52}$$

$$Y_{i2} = \delta g_{i2} + \alpha g_{\text{par(i)}} + \epsilon_{i2}; \tag{53}$$

where $\alpha = (\alpha_p + \alpha_m)/2$ is the average non-transmitted coefficient, and the residuals ϵ_{i1} and ϵ_{i2} are uncorrelated with the parent and offspring genotypes and have $\text{Var}(\epsilon_{i1}) = \text{Var}(\epsilon_{i2}) = \sigma_\epsilon^2$, and $\text{Corr}(\epsilon_{i2}, \epsilon_{i2}) = r$.

4.1 Without imputation

The phenotypes of the two siblings can be transformed into two orthogonal variables:

$$Y_{i1} - Y_{i2} = \delta(g_{i1} - g_{i2}) + \epsilon_{i1} - \epsilon_{i2}; \tag{54}$$

$$Y_{i1} + Y_{i2} = \delta(g_{i1} + g_{i2}) + 2\alpha g_{\text{par(i)}} + \epsilon_{i1} + \epsilon_{i2}. \tag{55}$$

The first variable, $Y_{i1} - Y_{i2}$, gives information on δ. The second variable gives information on a linear combination of δ and α that, when combined with the information on δ from the difference in phenotypes, can give an estimate of α.

By performing regression of differences between siblings' phenotypes onto differences in genotypes, one can estimate δ. Let $\hat{\delta}_\Delta$ be the resulting estimator. It can be shown that $\mathbb{E}[\hat{\delta}_\Delta] = \delta$ when $\eta_s = 0$; and

$$\text{Var}(\hat{\delta}_\Delta) = \frac{(1-r)\sigma_\epsilon^2}{nf(1-f)}. \tag{56}$$

By regression of $(Y_{i1} + Y_{i2})$ on $(g_{i1} + g_{i2})$ one obtains an estimate of $\delta + (4/3)\alpha$. Let this estimate be $\hat{a}$. It is trivial to show that $\text{Var}(\hat{a}) = (1+r)\sigma_\epsilon^2/(3nf(1-f))$. We can obtain an estimate of α as $\hat{\alpha}_\Delta = (3/4)(\hat{a} - \hat{\delta}_\Delta)$, with $\mathbb{E}[\hat{\alpha}_\Delta] = \alpha$. From this, we have that $\text{Var}(\hat{\alpha}_\Delta) = 3\sigma_\epsilon^2(2-r)/(8nf(1-f))$. We also have that $\text{Cov}(\hat{\alpha}_\Delta, \hat{\delta}_\Delta) = -3\text{Var}(\hat{\delta}_\Delta)/4 = -3(1-r)\sigma_\epsilon^2/(4nf(1-f))$.

4.2 With imputation

Given that $\eta_s = 0$, the results of Section 2 imply that least-squares and generalised least-squares regression of phenotype onto proband and imputed parental genotype gives an unbiased and consistent estimator of $(\delta, \alpha)^T$.

Let $\hat{g}_{\text{par}(i)}$ be the imputed parental genotype from one of the above methods — without IBD, with phased data and IBD, and with IBD but without phased data. From Lemma 1, we have that

$$\begin{bmatrix} \text{Var}(g_{i1}) & \text{Cov}(g_{i1}, g_{i2}) & \text{Cov}(g_{i1}, \hat{g}_{\text{par(i)}}) \\ \text{Cov}(g_{i1}, g_{i2}) & \text{Var}(g_{i2}) & \text{Cov}(g_{i2}, \hat{g}_{\text{par(i)}}) \\ \text{Cov}(g_{i1}, \hat{g}_{\text{par(i)}}) & \text{Cov}(g_{i2}, \hat{g}_{\text{par(i)}}) & \text{Var}(\hat{g}_{\text{par(i)}}) \end{bmatrix} = f(1-f) \begin{bmatrix} 2 & 1 & 2 \\ 1 & 2 & 2 \\ 2 & 2 & 4v \end{bmatrix}, \tag{57}$$

where v is the fraction of the variance in parental genotype captured by the imputation.

We compute the sampling variance of from n independent families with two siblings in each family, where

$$\hat{X}_i = \begin{bmatrix} g_{i1} & \hat{g}_{\text{par(i)}} \\ g_{i2} & \hat{g}_{\text{par(i)}} \end{bmatrix} \tag{58}$$

is the design matrix for family i, with g_{i1} the genotype of sibling 1 in family i, g_{i2} the genotype of sibling 2 in family i, and $\hat{g}_{\text{par(i)}}$ the imputed parental genotype for family i. For convenience in this computation, we assume that the genotypes have been mean-centred.

The generalised least-squares estimator is:

$$\hat{\theta} = \left(\sum_{i=1}^{n} \hat{X}_i^T \Sigma_i^{-1} \hat{X}_i \right)^{-1} \left(\sum_{i=1}^{n} \hat{X}_i^T \Sigma_i^{-1} Y_i \right). \tag{59}$$

Assuming that α^2 is negligible compared to the phenotypic variance (Section 2),

$$\text{Var}(\hat{\theta}) \approx \left(\sum_{i=1}^{n} \hat{X}_i^T \Sigma_i^{-1} \hat{X}_i \right)^{-1}. \tag{60}$$

We have that:

$$X_i^T \Sigma_i^{-1} X_i = \frac{1}{\sigma_\epsilon^2 (1-r^2)} \begin{bmatrix} g_{i1} & g_{i2} \\ \hat{g}_{\text{par(i)}} & \hat{g}_{\text{par(i)}} \end{bmatrix} \begin{bmatrix} 1 & -r \\ -r & 1 \end{bmatrix} \begin{bmatrix} g_{i1} & \hat{g}_{\text{par(i)}} \\ g_{i2} & \hat{g}_{\text{par(i)}} \end{bmatrix} \tag{61}$$

$$= \frac{1}{\sigma_\epsilon^2 (1-r^2)} \begin{bmatrix} g_{i1}^2 - 2r g_{i1} g_{i2} + g_{i2}^2 & \hat{g}_{\text{par(i)}}(g_{i1} - r(g_{i1} + g_{i2}) + g_{i2}) \\ \hat{g}_{\text{par(i)}}(g_{i1} - r(g_{i1} + g_{i2}) + g_{i2}) & 2(1-r)\hat{g}_{\text{par(i)}}^2 \end{bmatrix} \tag{62}$$

As the sample size increases,

$$\sum_{i=1}^{n} \hat{X}_i^T \Sigma_i^{-1} \hat{X}_i \to \frac{2nf(1-f)}{\sigma_\epsilon^2 (1-r^2)} \begin{bmatrix} 2-r & 2(1-r) \\ 2(1-r) & 4v(1-r) \end{bmatrix}. \tag{63}$$

Therefore,

$$\left(\sum_{i=1}^{n} \hat{X}_i^T \Sigma_i^{-1} \hat{X}_i\right)^{-1} \to \frac{\sigma_\epsilon^2(1+r)}{8[(2-r)v+r-1]nf(1-f)} \begin{bmatrix} 4v(1-r) & -2(1-r) \\ -2(1-r) & (2-r) \end{bmatrix}. \tag{64}$$

Using the above, we can derive the approximate large-sample variance of the generalised least squares estimator using different imputation methods:

method	v	$\text{Var}(\hat{\delta})^1$	$\text{Var}(\hat{\alpha})^1$
no imputation	-	$(1-r)$	$(3/8)(2-r)$
IBD and unphased	$3/4 - f(1-f)/8$	$\frac{(1-r^2)(3-f(1-f)/2)}{2[2+r-(1-r/2)f(1-f)]}$	$\frac{(1+r)(2-r)}{2[2+r-(1-r/2)f(1-f)]}$
IBD and phased	$3/4$	$\frac{3(1-r^2)}{2(2+r)}$	$\frac{(1+r)(2-r)}{2(2+r)}$
complete data	1	$\frac{1-r^2}{2}$	$\frac{(1+r)(2-r)}{8}$

Supplementary Note Table 6: The sampling variance of estimators of the direct effect and average non-transmitted coefficient using no imputation, imputation using IBD and un-phased data, using phased data and IBD, and using complete data (both maternal and paternal genotype observed). These are given for a sample of n independent sibling pairs with a correlation of r between their residuals. [1]The sampling variance is given as the factor that multiplies $\sigma_\epsilon^2/(nf(1-f))$.

4.3 Asymmetrical sibling pairs

Here we relax the assumption that $\eta_s = 0$ and allow for effects to differ between two types of siblings (such as older or younger, or male or female):

$$Y_{i1} = \delta_1 g_{i1} + \eta_{s1} g_{i2} + \alpha_1 g_{\text{par}(i)} + \epsilon_{i1}, \tag{65}$$

$$Y_{i2} = \delta_2 g_{i2} + \eta_{s2} g_{i1} + \alpha_2 g_{\text{par}(i)} + \epsilon_{i2}. \tag{66}$$

The model can be transformed into two approximately uncorrelated variables:

$$Y_{+i} = (\delta_1 + \eta_{s2}) g_{i1} + (\delta_2 + \eta_{s1}) g_{i2} + (\alpha_1 + \alpha_2) g_{\text{par}(i)} + \epsilon_{i1} + \epsilon_{i2}, \tag{67}$$

$$Y_{-i} = (\delta_1 - \eta_{s2}) g_{i1} - (\delta_2 - \eta_{s1}) g_{i2} + (\alpha_1 - \alpha_2) g_{\text{par}(i)} + \epsilon_{i2} - \epsilon_{i2}. \tag{68}$$

As long as the genotypes at the SNP account for only a small fraction of the variance of the phenotypic variance, and provided that $\text{Var}(\epsilon_{i1}) \approx \text{Var}(\epsilon_{i2})$, we have that $\text{Cov}(Y_{+i}, Y_{-i}) \approx 0$. Let us assume that $\text{Var}(\epsilon_{i1}) = \text{Var}(\epsilon_{i2}) = \sigma_\epsilon^2$, and therefore $\text{Var}(\epsilon_{i1} + \epsilon_{i2}) = 2(1+r)\sigma_\epsilon^2$ and $\text{Var}(\epsilon_{i1} - \epsilon_i) = 2(1-r)\sigma_\epsilon^2$. Further, let $\theta_1 = [\delta_1, \eta_{s1}, \alpha_1]^T$ and $\theta_2 = [\eta_{s2}, \delta_2, \alpha_2]^T$, and let

$$\hat{X}_i = \begin{bmatrix} g_{i1} & g_{i2} & \hat{g}_{\text{par(i)}} \\ g_{i2} & g_{i1} & \hat{g}_{\text{par(i)}} \end{bmatrix}, \tag{69}$$

where $\hat{g}_{\text{par(i)}}$ has been imputed from the siblings' genotypes and phased data. By the results in Section 2, we have that the regression $Y_{+i} \sim \hat{X}_i$ gives an unbiased and consistent estimator of $\theta_+ = \theta_1 + \theta_2$; and the regression $Y_{-i} \sim \hat{X}_i$ gives an unbiased and consistent estimator of $\theta_- = \theta_1 - \theta_2$. We now compute the variance of the estimator from n independent families. Assuming that the variance explained by the SNP is a small fraction of the phenotypic variance:

$$\text{Var}(\hat{\theta}_+) \approx \frac{2(1+r)\sigma_\epsilon^2}{n}\text{Var}(\hat{X}_i)^{-1}; \ \text{Var}(\hat{\theta}_-) \approx \frac{2(1-r)\sigma_\epsilon^2}{n}\text{Var}(\hat{X}_i)^{-1}. \tag{70}$$

From the above results, we have that

$$\text{Var}(\hat{X}_i) = f(1-f)\begin{bmatrix} 2 & 1 & 2 \\ 1 & 2 & 2 \\ 2 & 2 & 3 \end{bmatrix}. \tag{71}$$

Therefore,

$$\text{Var}(\hat{X}_i)^{-1} = \frac{1}{f(1-f)}\begin{bmatrix} 2 & 1 & -2 \\ 1 & 2 & -2 \\ -2 & -2 & 3 \end{bmatrix}. \tag{72}$$

By combining the results of these two approximately uncorrelated regressions, and then through linear transformation, we can estimate average and difference parameters for the effects on the two sibling types. We have that

$$\theta = \begin{bmatrix} \delta = (\delta_1 + \delta_2)/2 \\ \eta_s = (\eta_{s1} + \eta_{s2})/2 \\ \alpha = (\alpha_1 + \alpha_2)/2 \\ \delta_- = \delta_1 - \delta_2 \\ \eta_{s-} = \eta_{s1} - \eta_{s2} \\ \alpha_- = \alpha_1 - \alpha_2 \end{bmatrix} = A\begin{bmatrix} \theta_- \\ \theta_+ \end{bmatrix}, \tag{73}$$

where

$$A = \begin{bmatrix} 1/4 & -1/4 & 0 & 1/4 & 1/4 & 0 \\ -1/4 & 1/4 & 0 & 1/4 & 1/4 & 0 \\ -1/8 & 1/8 & 0 & 1/8 & 1/8 & 1/2 \\ 1/2 & 1/2 & 0 & 1/2 & -1/2 & 0 \\ 1/2 & 1/2 & 0 & -1/2 & 1/2 & 0 \\ 1/4 & 1/4 & 1 & -1/4 & 1/4 & 0 \end{bmatrix} = \begin{bmatrix} A_{11} & A_{12} \\ A_{21} & A_{22} \end{bmatrix}, \tag{74}$$

where A_{ij} are all $[3 \times 3]$ matrices. We then have that

$$\begin{bmatrix} \hat{\delta} \\ \hat{\eta}_s \\ \hat{\alpha} \end{bmatrix} = A_{11}\hat{\theta}_- + A_{12}\hat{\theta}_+ \tag{75}$$

Based on the fact that $\hat{\theta}_-$ and $\hat{\theta}_+$ are approximately uncorrelated with variances given above, it can be shown that

$$\text{Var}\left(\begin{bmatrix}\hat{\delta}\\ \hat{\eta}_s\\ \hat{\alpha}\end{bmatrix}\right) \approx \frac{\sigma_\epsilon^2}{2nf(1-f)}\begin{bmatrix}2+r & 1+2r & -(3/2+r)\\ 1+2r & 2+r & -(1+3r/2)\\ -(3/2+r) & -(1+3r/2) & 3/2+5r/4\end{bmatrix}. \tag{76}$$

If we condition on $\eta_s = 0$, then the estimate (δ, α) is

$$\begin{bmatrix}\hat{\delta}_{\eta_s=0}\\ \hat{\alpha}_{\eta_s=0}\end{bmatrix} = \begin{bmatrix}\hat{\delta}\\ \hat{\alpha}\end{bmatrix} + \begin{bmatrix}\frac{-(1+2r)}{2+r}\\ \frac{1+3r/2}{2+r}\end{bmatrix}\hat{\eta}_s, \tag{77}$$

with variance

$$\text{Var}\left(\begin{bmatrix}\hat{\delta}\\ \hat{\alpha}\end{bmatrix}\right) \approx \frac{\sigma_\epsilon^2(1+r)}{2(2+r)nf(1-f)}\begin{bmatrix}3(1-r) & -2(1-r)\\ -2(1-r) & (2-r)\end{bmatrix}, \tag{78}$$

as derived in Equation 64 with $v = 3/4$ for the generalised least-squares estimator assuming that $\eta_s = 0$. The effective sample size for estimation of δ is $(2+r)^2/((3(1-r^2))$ times higher when assuming $\eta_s = 0$ compared to also estimating η_s; i.e., at least 4/3 times higher for $r \geq 0$. This shows that if $\eta_s \neq 0$, assuming that $\eta_s = 0$ gives a more precise estimator of direct and average parental effects at the cost of some bias. We note, however, that the bias is less than when not performing imputation. Without performing imputation, the estimate of the direct effect has bias $-\eta_s$, which is larger than with imputation, $\frac{-(1+2r)}{2+r}\eta_s$, being only $-\eta_s/2$ when $r = 0$, for example.

Similarly, the variance of the estimates of the difference parameters, which are uncorrelated with the estimates of the average parameters, can be shown to be

$$\text{Var}\left(\begin{bmatrix}\hat{\delta}_-\\ \hat{\eta}_{s-}\\ \hat{\alpha}_-\end{bmatrix}\right) \approx \frac{\sigma_\epsilon^2}{2nf(1-f)}\begin{bmatrix}8-4r & 4-8r & -6+4r\\ 4-8r & 8-4r & -4+6r\\ -6+4r & -4+6r & 6-5r\end{bmatrix}. \tag{79}$$

5 One parent missing

5.1 Imputation without phased data

We impute the missing parental genotype as the expectation given the observed proband and parent genotypes. Assuming that the father's genotype is missing, this is

$$\hat{g}_{\text{p(i)}} = \mathbb{E}[g_{\text{p(i)}}|g_i, g_{\text{m(i)}}] \tag{80}$$

$$= \frac{2[f(1-f)\mathbb{P}(g_i|g_{\text{m(i)}}, g_{\text{p(i)}}=1) + f^2\mathbb{P}(g_i|g_{\text{m(i)}}, g_{\text{p(i)}}=2)]}{(1-f)^2\mathbb{P}(g_i|g_{\text{m(i)}}, g_{\text{p(i)}}=0) + 2f(1-f)\mathbb{P}(g_i|g_{\text{m(i)}}, g_{\text{p(i)}}=1) + f^2\mathbb{P}(g_i|g_{\text{m(i)}}, g_{\text{p(i)}}=2)}, \tag{81}$$

which is derived from application of Bayes' Rule.

By applying the Laws of Mendelian Inheritance to compute the above probabilities, one can derive that:

		$g_{\mathrm{m(i)}}$		
		0	1	2
	0	f	f	-
g_i	1	$1+f$	$2f$	f
	2	-	$1+f$	$1+f$

Supplementary Note Table 7: $\mathbb{E}[g_{\mathrm{p(i)}}|g_i, g_{\mathrm{m(i)}}]$

Note that it is impossible for a parent to have two copies of an allele and for the offspring to inherit zero copies, without mutation. (We ignore the possibility of genotyping error here.)

5.1.1 Multiple siblings

We generalise the above approach to perform imputation when two or more full sibling offspring of the observed parent are genotyped. As outlined above for imputation from siblings without genotyped parents, imputation from three or more siblings can be reduced to imputation from a single sibling pair in either IBD state 0, 1, or 2. We therefore give the values for the imputed genotype of the missing father given observed maternal genotype, $g_{m(i)}$, and observations on two sibling genotype (g_{i1}, g_{i2}), and the IBD state of the siblings.

We apply Bayes' Rule to derive the probability of the paternal genotype given the observed genotypes and IBD state of the sibling pair:

$$\mathbb{P}(g_{p(i)}|g_{i1}, g_{i2}, g_{m(i)}, \mathrm{IBD}=t) = \frac{\mathbb{P}(g_{i1}, g_{i2}|g_{p(i)}, g_{m(i)}, \mathrm{IBD}=t)\mathbb{P}(g_{p(i)}|g_{m(i)}, \mathrm{IBD}=t)}{\mathbb{P}(g_{i1}, g_{i2}|g_{m(i)}, \mathrm{IBD}=t)} \tag{82}$$

$$= \frac{\mathbb{P}(g_{i1}, g_{i2}|g_{m(i)}, g_{p(i)}, \mathrm{IBD}=t)\mathbb{P}(g_{p(i)})}{\sum_{g_{p(i)}\in\{0,1,2\}} \mathbb{P}(g_{i1}, g_{i2}|g_{m(i)}, g_{p(i)}, \mathrm{IBD}=t)\mathbb{P}(g_{p(i)})} \tag{83}$$

From this, we can derive the expectation of the paternal genotype given the observed genotypes and IBD state of the siblings. We give tables for these values in Appendix B.

5.2 Imputation with phased data

As outlined in 3.2.1, when multiple siblings are present, the imputation problem can be reduced to a single sibling pair either in IBD0, IBD1, or IBD2. When a single offspring is present, this is equivalent to a sibling pair in IBD2.

There are therefore three cases:

1. We have siblings in IBD0: the imputed parental genotype is the sum of the siblings' genotypes minus the observed parent's genotype;

2. We have a sibling pair in IBD1 and no pairs in IBD0: in this case, one allele is shared between the two siblings in IBD1. We first find that allele using the phased haplotype surrounding the SNP. If that allele is shared between the sibling pair and the observed parent, the missing parent's genotype is exactly the sum of the alleles unshared between the sibling pair. If that allele is not shared between the sibling pair and the observed parent, the imputed genotype is the sum of the shared allele and the allele frequency;

3. All siblings are in IBD2: in this case, all the siblings have the same genotype. We determine which allele in the siblings is shared with the observed parent, using the phased haplotype if both sibling and observed parent genotype are heterozygous. The imputed genotype is the sum of the allele in the siblings not shared with the observed parent and the allele frequency.

When a pair of individuals is IBD1 and both are heterozygous at a SNP, we determine which allele is shared by determining which phased haplotype in that region is shared. We use a window of 100 SNPs around the target SNP, and we determine that a haplotype is shared if there is perfect agreement between haplotypes in that window.

5.3 Association analysis

Consider a sample of n independent families with one parent and one child genotyped. We assume the genotyped parent is the mother for all families for notational convenience.

The phenotype of the proband from family i can be expressed as

$$Y_i = \delta g_i + \alpha_p g_{\text{p(i)}} + \alpha_m g_{\text{m(i)}} + \epsilon_i, \tag{84}$$

for some mean-zero ϵ_i such that $\text{Cov}(g_i, \epsilon_i) = \text{Cov}(g_{\text{p(i)}}, \epsilon_i) = \text{Cov}(g_{\text{m(i)}}, \epsilon_i) = 0$, and $\text{Var}(\epsilon_i) = \sigma_\epsilon^2$ for all i.

Let $[\hat{X}_p]_i = [g\,, g_{m(i)}\,, \hat{g}_{p(i)}]$, where $[\hat{X}_p]_i$ is the ith row of $\hat{X}_p$, and let $[Y]_i = Y_i$ be the ith element of the phenotype column vector Y. We consider an estimator formed by regression of Y onto $\hat{X}_p$: $\hat{\theta}_p = (\hat{X}_p^T \hat{X})^{-1} \hat{X}_p^T Y$. The imputed parental genotype is the conditional expectation given the proband and maternal genotype: $\hat{g}_{\text{p(i)}} = \mathbb{E}[g_{\text{p(i)}} | g_i, g_{\text{m(i)}}]$. This means we can apply the theory in Section 2 to derive that $\mathbb{E}[\hat{\theta}_p] = [\delta, \alpha_p, \alpha_m]^T$ and $\lim_{n\to\infty} \hat{\theta}_p = [\delta, \alpha_p, \alpha_m]^T$.

We now derive the sampling variance of $\hat{\theta}_p$. From Section 2.3, we have that

$$\text{Var}(\hat{\theta}_p) = [\sigma_\epsilon^2 + (\text{Var}(g_{p(i)}) - \text{Var}(\hat{g}_{p(i)}))\alpha_p^2](\hat{X}_p^T \hat{X}_p)^{-1} \tag{85}$$

As $n \to \infty$,

$$\text{Var}(\hat{\theta}_p) \to \frac{\sigma_\epsilon^2 + (\text{Var}(g_{p(i)}) - \text{Var}(\hat{g}_{p(i)}))\alpha_p^2}{n} \text{Var}(\hat{X}_p)^{-1} = \frac{\sigma_\epsilon^2}{n} \text{Var}(\hat{X}_p)^{-1} + \text{O}(\alpha_p^2). \tag{86}$$

For typical analysis of individual SNPs, the $\text{O}(\alpha_p^2)$ term is negligible and can be ignored.

We first derive the variance of the estimator when imputing with phased data. Since we always recover one of the missing father's alleles, we have that $\text{Var}(\hat{g}_{p(i)}) = f(1-f)$, and therefore, by Lemma 1,

$$\text{Var}(\hat{X}_p) = f(1-f)\begin{bmatrix} 2 & 1 & 1 \\ 1 & 2 & 0 \\ 1 & 0 & 1 \end{bmatrix}; \Rightarrow \text{Var}(\hat{\theta}_p) \to \frac{\sigma_\epsilon^2 + f(1-f)\alpha_p^2}{nf(1-f)} \begin{bmatrix} 2 & -1 & -2 \\ -1 & 1 & 1 \\ -2 & 1 & 3 \end{bmatrix}. \tag{87}$$

This shows that the variance for the estimator of direct effects is twice that of the estimator with complete observations of genotypes (plus an $\text{O}(\alpha_p^2)$ term).

To derive $\text{Var}(\hat{g}_{\text{p(i)}})$ when imputing without phased data, we first derive the joint probabilities of the observed genotypes using Bayes' Rule and the Laws of Mendelian Inheritance:

		$g_{\text{m(i)}}$		
		0	1	2
	0	$(1-f)^3$	$f(1-f)^2$	0
g_i	1	$f(1-f)^2$	$f(1-f)$	$f^2(1-f)$
	2	0	$f^2(1-f)$	f^3

Supplementary Note Table 8: $\mathbb{P}(g_i, g_{\text{m(i)}})$

From this, we can compute that $\text{Var}(\hat{g}_{\text{p(i)}}) = f(1-f)[1-f(1-f)]$. By application of Lemma 1, we have that

$$\text{Var}(\hat{X}_p) = f(1-f)\begin{bmatrix} 2 & 1 & 1 \\ 1 & 2 & 0 \\ 1 & 0 & 1-f(1-f) \end{bmatrix}; \tag{88}$$

and therefore

$$\text{Var}(\hat{\theta}_p) \to \frac{\sigma_\epsilon^2 + f(1-f)[1+f(1-f)]\alpha_p^2}{nf(1-f)[1-3f(1-f)]} \begin{bmatrix} 2-2f(1-f) & -(1-f(1-f)) & -2 \\ -(1-f(1-f)) & 1-2f(1-f) & 1 \\ -2 & 1 & 3 \end{bmatrix}. \tag{89}$$

Let $\hat{\delta}_p$ be the resulting estimator of δ, then

$$\text{Var}(\hat{\delta}_p) \to \frac{[2-2f(1-f)]\sigma_\epsilon^2}{[1-3f(1-f)]nf(1-f)} + \text{O}(\alpha_p^2) \tag{90}$$

This can be compared to the variance of the estimator of delta with both parental genotypes observed, $\hat{\delta}_{po}$: $\text{Var}(\hat{\delta}_{po}) = \sigma_\epsilon^2(nf(1-f))^{-1}$; and

$$\frac{\text{Var}(\hat{\delta}_{po})}{\text{Var}(\hat{\delta}_p)} = \frac{1-3f(1-f)}{2-2f(1-f)} + \text{O}(\alpha_p^2). \tag{91}$$

Without phased data, the penalty relative to using fully observed parental genotypes increases with the heterozygosity due to the fact that when both observed parent and child genotypes are heterozygous, the allele inherited by the child from the observed parent cannot be determined, so an average over the two possible inheritance patterns is taken as the imputation.

6 Multivariate Meta-analysis

Consider a parameter vector θ and independent observations $z_i \sim \mathcal{N}(A_i\theta, \Sigma_i)$ for $i = 1, \ldots, k$, then it can be shown that the MLE for θ is

$$\hat{\theta} = \left(\sum_{i=1}^{k} A_i^T \Sigma_i^{-1} A_i \right)^{-1} \left(\sum_{i=1}^{k} A_i^T \Sigma_i^{-1} z_i \right), \tag{92}$$

with $\mathbb{E}[\hat{\theta}] = \theta$ and

$$\text{Var}(\hat{\theta}) = \left(\sum_{i=1}^{k} A_i^T \Sigma_i^{-1} A_i \right)^{-1}. \tag{93}$$

Note that this assumes that $\sum_{i=1}^{k} A_i^T \Sigma_i^{-1} A_i$ is invertible.

Consider estimating parameters in the model

$$Y_i = \delta g_i + \alpha_p g_{p(i)} + \alpha_m g_{m(i)} + \epsilon_i, \tag{94}$$

where ϵ_i is uncorrelated with g_i, $g_{p(i)}$, and $g_{m(i)}$. We assume that indirect effects from siblings are zero, $\eta_s = 0$. We consider estimating $\theta = [\delta, \alpha_p, \alpha_m]^T$ using different samples with different observations of sibling and parental alleles.

We analyse theoretically a simple scenario where we combine results from a trio GWAS that regresses phenotype onto proband genotype and the sum of maternal and paternal genotypes, giving estimates of direct and average non-transmitted coefficients. Let $\theta = [\delta, \alpha]^T$ and let n_0 be the number of independent individuals with both parents genotyped used in the trio GWAS, and let n_1 be the number of independent individuals used in the standard GWAS. Then we have that

$$z_0 \sim \mathcal{N}\left(\theta, \frac{\sigma_\epsilon^2}{n_0 2f(1-f)} \begin{bmatrix} 1 & 1 \\ 1 & 2 \end{bmatrix}^{-1} \right); \tag{95}$$

and

$$z_1 \sim \mathcal{N}\left(\begin{bmatrix} 1 \\ 1 \end{bmatrix}^T \theta, \frac{\sigma_\epsilon^2}{n_1 2f(1-f)} \right). \tag{96}$$

We therefore have that

$$\text{Var}(\hat{\theta}) = \left(\frac{n_0 2f(1-f)}{\sigma_\epsilon^2} \begin{bmatrix} 1 & 1 \\ 1 & 2 \end{bmatrix} + \frac{n_1 2f(1-f)}{\sigma_\epsilon^2} \begin{bmatrix} 1 & 1 \\ 1 & 1 \end{bmatrix} \right)^{-1} \tag{97}$$

$$= \frac{\sigma_\epsilon^2}{2f(1-f)} \begin{bmatrix} n_0+n_1 & n_0+n_1 \\ n_0+n_1 & 2n_0+n_1 \end{bmatrix}^{-1} \tag{98}$$

$$= \frac{\sigma_\epsilon^2}{2f(1-f)} \frac{1}{n_0(n_0+n_1)} \begin{bmatrix} 2n_0+n_1 & -(n_0+n_1) \\ -(n_0+n_1) & n_0+n_1 \end{bmatrix}. \tag{99}$$

We therefore have that

$$\text{Var}(\hat{\delta}) = \frac{\sigma_\epsilon^2}{2f(1-f)} \left(\frac{1}{n_0} + \frac{1}{n_0+n_1} \right). \tag{100}$$

We can compare this to the variance of the estimator of direct effects using only the n_0 trios, $\hat{\delta}_0$:

$$\frac{\text{Var}(\hat{\delta}_0)}{\text{Var}(\hat{\delta})} = 1 + \frac{n_1}{2n_0+n_1} \to 2 \text{ as } \frac{n_1}{n_0} \to \infty. \tag{101}$$

Similarly, if we consider combining a sample of n_0 sibling pairs where we have imputed the sum of maternal and paternal genotypes using phased data with a sample of n_1 singletons, it can be shown that, assuming the correlation between siblings' residuals is zero ($r = 0$),

$$\text{Var}(\hat{\theta}) = \frac{\sigma_\epsilon^2}{2f(1-f)} \frac{1}{n_0(2n_0+n_1)} \begin{bmatrix} 3n_0+n_1 & -(2n_0+n_1) \\ -(2n_0+n_1) & 2n_0+n_1 \end{bmatrix}. \tag{102}$$

We can compare this to the variance of the estimator of direct effects using only the n_0 sibling pairs, $\hat{\delta}_0$:

$$\frac{\text{Var}(\hat{\delta}_0)}{\text{Var}(\hat{\delta})} = 1 + \frac{n_1}{2(3n_0+n_1)} \to 1.5 \text{ as } \frac{n_1}{n_0} \to \infty. \tag{103}$$

7 Effect of population structure

We analyse estimation of direct effects and NTCs using imputed parental genotypes in a structured population. We consider a population divided into K subpopulations, where within each subpopulation, there is random-mating, and there is no migration between subpopulations. Let g_{kij} be the genotype of sibling j in family i in subpopulation $k = 1, \ldots, K$. We denote the allele frequency in subpopulation k as f_k, and the overall allele frequency in the population, $f = \mathbb{E}_k[f_k]$, where the expectation is taken over the k subpopulations. The measure of population structure relevant for the theoretical results in this section is $F_{ST} = \frac{\text{Var}_k(f_k)}{f(1-f)}$, where $\text{Var}_k(f_k)$ is the variance of allele frequencies across subpopulations.

If the subpopulation memberships of each family were known, then the imputation could use the subpopulation specific allele frequencies. However, a more realistic scenario is population

structure that is unknown, so that imputation proceeds assuming random-mating in the overall population, i.e. using the allele frequencies of the overall population.

The imputation from a sibling pair using phased data and IBD is therefore:

$$\hat{g}_{k\text{par}(i)} = \begin{cases} g_{ki1} + g_{ki2} = g_{k\text{par}(i)}, & \text{if IBD} = 0 \\ g_{ki1} + g^a_{ki2} + f, & \text{if IBD} = 1 \\ g_{ki1} + 2f, & \text{if IBD} = 2, \end{cases} \tag{104}$$

where $a \in \{m, p\}$ is such that g^a_{ki2} is not IBD with the alleles inherited by sibling 1 in family i in subpopulation k, and $g_{k\text{par}(i)}$ is the sum of maternal and paternal genotypes in family i from subpopulation k. From this, we derive that $\mathbb{E}[\hat{g}_{k\text{par}(i)}] = 3f_k + f$, which implies that the imputation is biased when $f_k \neq f$. We therefore cannot directly carry over theoretical results from Section 2 that show estimates are unbiased and consistent when using parental genotypes imputed using the overall population frequency when $F_{ST} > 0$.

In order to determine what bias, if any, imputation using overall allele frequencies in a structured population introduces, the following results are useful. First, the variance of the genotype in the overall population, $\text{Var}(g)$, which we compute using the Law of Total Variance:

$$\text{Var}(g) = \mathbb{E}_k[\text{Var}(g_{kij})] + \text{Var}_k(\mathbb{E}[g_{kij}]); \tag{105}$$
$$= \mathbb{E}_k[2f_k(1 - f_k)] + \text{Var}_k(2f_k); \tag{106}$$
$$= 2f - 2\mathbb{E}[f_k^2] + 4\text{Var}_k(f_k); \tag{107}$$
$$= 2f(1 - f) + 2\text{Var}(f_k); \tag{108}$$
$$= 2f(1 - f)\left[1 + \frac{\text{Var}(f_k)}{f(1 - f)}\right]; \tag{109}$$
$$= 2f(1 - f)[1 + F_{ST}]. \tag{110}$$

Following a similar procedure, we compute the variance of the combined parental genotype, $\text{Var}(g_{\text{par}})$, in the overall population:

$$\text{Var}(g_{\text{par}}) = \mathbb{E}_k[\text{Var}(g_{k\text{par}(i)})] + \text{Var}_k(\mathbb{E}[g_{k\text{par}(i)}]); \tag{111}$$
$$= \mathbb{E}_k[4f_k(1 - f_k)] + \text{Var}_k(4f_k); \tag{112}$$
$$= 4f(1 - f)[1 + 3F_{ST}]. \tag{113}$$

The covariance between offspring and parent genotype, $\text{Cov}(g, g_{\text{par}})$, in the overall population is:

$$\text{Cov}(g, g_{\text{par}}) = \mathbb{E}_k[\text{Cov}(g_{kij}, g_{k\text{par}(i)})] + \text{Cov}_k(\mathbb{E}[g_{kij}], \mathbb{E}[g_{k\text{par}(i)}]); \tag{114}$$
$$= \mathbb{E}_k[2f_k(1 - f_k)] + \text{Cov}_k(2f_k, 4f_k); \tag{115}$$
$$= 2f(1 - f)[1 + 3F_{ST}]. \tag{116}$$

While there is random-mating within each subpopulation, so that maternal and paternal genotypes are uncorrelated, this is not true in the overall population when $F_{ST} > 0$:

$$\begin{aligned}\mathrm{Cov}(g_m, g_p) &= \mathbb{E}_k[\mathrm{Cov}(g_{k\mathrm{m}(i)}, g_{k\mathrm{p}(i)})] + \mathrm{Cov}_k(\mathbb{E}[g_{k\mathrm{m}(i)}], \mathbb{E}[g_{k\mathrm{p}(i)}]); &(117)\\ &= \mathrm{Cov}_k(2f_k, 2f_k); &(118)\\ &= 4f(1-f)F_{ST}. &(119)\end{aligned}$$

7.1 Imputation from siblings

Here we analyse estimating direct and average non-transmitted coefficients in a structured population when parental genotypes are imputed from sibling pair genotypes using the allele frequency in the overall population, as defined in (104). The phenotype model is

$$Y_{kij} = \delta g_{kij} + \alpha g_{k\mathrm{par}(i)} + \epsilon_{kij}, \tag{120}$$

where ϵ_{kij} is uncorrelated with both proband and parental genotype. Let Y_k be the phenotype vector in subpopulation k, and let $\hat{X}_k = [g_{kij},\ \hat{g}_{k\mathrm{par}(i)}]$ be the corresponding matrix of proband and parental genotypes imputed from sibling genotypes and phased data using the overall allele frequency as in (104). We examine least-squares estimation of $\theta = [\delta, \alpha]^T$ in the overall population:

$$\hat{\theta} = (\hat{X}^T\hat{X})^{-1}\hat{X}^T Y, \text{ where } \hat{X} = \begin{bmatrix} X_1 \\ X_2 \\ \vdots \\ X_K \end{bmatrix} \text{ and } \hat{Y} = \begin{bmatrix} Y_1 \\ Y_2 \\ \vdots \\ Y_K \end{bmatrix}. \tag{121}$$

As the overall sample size goes to infinity,

$$\hat{\theta} \to \begin{bmatrix} \mathrm{Var}(g) & \mathrm{Cov}(g, \hat{g}_{\mathrm{par}}) \\ \mathrm{Cov}(g, \hat{g}_{\mathrm{par}}) & \mathrm{Var}(\hat{g}_{\mathrm{par}}) \end{bmatrix}^{-1} \begin{bmatrix} \mathrm{Var}(g) & \mathrm{Cov}(g, g_{\mathrm{par}}) \\ \mathrm{Cov}(\hat{g}_{\mathrm{par}}, g) & \mathrm{Cov}(\hat{g}_{\mathrm{par}}, g_{\mathrm{par}}) \end{bmatrix} \theta. \tag{122}$$

To compute the limit, we need to compute $\mathrm{Cov}(g, \hat{g}_{\mathrm{par}})$, $\mathrm{Cov}(\hat{g}_{\mathrm{par}}, g_{\mathrm{par}})$, and $\mathrm{Var}(\hat{g}_{\mathrm{par}})$. We begin with $\mathrm{Var}(\hat{g}_{\mathrm{par}})$:

$$\mathrm{Var}(\hat{g}_{\mathrm{par}}) = \mathbb{E}_k[\mathrm{Var}(\hat{g}_{k\mathrm{par}(i)})] + \mathrm{Var}_k(3f_k + f). \tag{123}$$

To compute $\mathrm{Var}(\hat{g}_{k\mathrm{par}(i)})$, we condition on the IBD state of the siblings:

$$\mathrm{Var}(\hat{g}_{k\mathrm{par}(i)}) = \mathbb{E}[\mathrm{Var}(\hat{g}_{k\mathrm{par}(i)}|\mathrm{IBD})] + \mathrm{Var}(\mathbb{E}[\hat{g}_{k\mathrm{par}(i)}|\mathrm{IBD}]). \tag{124}$$

As in the random-mating population, $\mathbb{E}[\mathrm{Var}(\hat{g}_{k\mathrm{par}(i)}|\mathrm{IBD})] = 3f_k(1-f_k)$. However, unlike in the random-mating population, the expectation of the imputed parental genotype depends upon the IBD state, since that determines how many alleles we impute with the overall population allele frequency, which differs from the allele frequency in each subpopulation. Since $\mathbb{E}[\hat{g}_{k\mathrm{par}(i)}] =$

$3f_k + f$, which is also the expectation when IBD=1, and the deviation from the expectation for IBD=0 is $(f_k - f)$ and for IBD=2 is $(f - f_k)$, we have

$$\mathrm{Var}(\mathbb{E}[\hat{g}_{k\mathrm{par}(i)}|\mathrm{IBD}]) = \frac{1}{4}(f_k - f)^2 + \frac{1}{4}(f - f_k)^2 = \frac{(f_k - f)^2}{2}. \tag{125}$$

Therefore,

$$\begin{aligned}\mathrm{Var}(\hat{g}_{\mathrm{par}}) &= \mathbb{E}_k[3f_k(1-f_k) + (f_k - f)^2/2] + 9\mathrm{Var}_k(f_k) &(126)\\ &= 3f(1-f)[1 + (13/6)F_{ST}]. &(127)\end{aligned}$$

We now compute $\mathrm{Cov}(g, \hat{g}_{\mathrm{par}})$:

$$\begin{aligned}\mathrm{Cov}(g, \hat{g}_{\mathrm{par}}) &= \mathbb{E}_k[\mathrm{Cov}(g_{kij}, \hat{g}_{k\mathrm{par}(i)})] + \mathrm{Cov}_k(2f_k, 3f_k + f); &(128)\\ &= \mathbb{E}_k[2f_k(1-f_k)] + 6\mathrm{Var}_k(f_k); &(129)\\ &= 2f(1-f)[1 + 2F_{ST}]; &(130)\end{aligned}$$

where we have used the fact that $\mathrm{Cov}(g_{kij}, \hat{g}_{k\mathrm{par}(i)}) = 2f_k(1-f_k)$ within each subpopulation.

We now compute $\mathrm{Cov}(\hat{g}_{\mathrm{par}}, g_{\mathrm{par}})$:

$$\begin{aligned}\mathrm{Cov}(\hat{g}_{\mathrm{par}}, g_{\mathrm{par}}) &= \mathbb{E}_k[\mathrm{Cov}(\hat{g}_{k\mathrm{par}(i)}, g_{k\mathrm{par}(i)})] + \mathrm{Cov}_k(3f_k + f, 4f_k); &(131)\\ &= \mathbb{E}_k[3f_k(1-f_k)] + 12\mathrm{Var}_k(f_k); &(132)\\ &= 3f(1-f)[1 + 3F_{ST}]; &(133)\end{aligned}$$

where we have used the fact that $\mathrm{Cov}(\hat{g}_{k\mathrm{par}(i)}, g_{k\mathrm{par}(i)}) = 3f_k(1-f_k)$ within each subpopulation. We can now compute the limit of $\hat{\theta}$ as the overall sample goes to infinity:

$$\hat{\theta} \to \begin{bmatrix} 2(1+F_{ST}) & 2(1+2F_{ST}) \\ 2(1+2F_{ST}) & 3(1+(13/6)F_{ST}) \end{bmatrix}^{-1} \begin{bmatrix} 2(1+F_{ST}) & 2(1+3F_{ST}) \\ 2(1+2F_{ST}) & 3(1+3F_{ST}) \end{bmatrix} \theta. \tag{134}$$

After some algebra, we obtain

$$\hat{\theta} \to \begin{bmatrix} \delta + b\alpha \\ (1+a)\alpha \end{bmatrix}; \tag{135}$$

where

$$b = \frac{F_{ST}(1+3F_{ST})}{2(1-F_{ST})(1+2F_{ST}) + F_{ST}(1+F_{ST})}; \text{ and} \tag{136}$$

$$a = \frac{F_{ST}(1-3F_{ST})}{2(1-F_{ST})(1+2F_{ST}) + F_{ST}(1+F_{ST})}. \tag{137}$$

When there is little differentiation at the locus and F_{ST} is small,

$$\hat{\theta} \to \begin{bmatrix} \delta + b\alpha \\ (1+a)\alpha \end{bmatrix} \approx \begin{bmatrix} \delta + (F_{ST}/2)\alpha \\ (1+(F_{ST}/2))\alpha \end{bmatrix}, \tag{138}$$

The imputation therefore leads to biased estimates of both δ and α in proportion to the F_{ST} at the locus. This is due to the fact that the expectation of the imputed parental genotype changes with the IBD state of the siblings, leading to excess variance in the imputed parental genotype that is correlated with subpopulation membership. Without the change in expectation with IBD state, the variance of the imputed parental genotype would be $3f(1-f)[1+2F_{ST}]$ rather than $3f(1-f)[1+(13/6)F_{ST}]$. It is straightforward to show that, if this was the case, then estimator for δ would be consistent.

7.2 Fixing the bias

The above analysis suggests that the bias derives from the fact that the expectation of the imputed parental genotype varies across IBD state of the siblings. If one performed separate regressions for siblings in different IBD states, this variation in the expectation of the imputed parental genotype would no longer be relevant. We therefore propose an estimator for δ that is robust to population structure:

1. Perform a regression of phenotype onto proband genotype and imputed parental genotype for siblings with IBD=0. Call this $\hat{\hat{\delta}}_0$
2. Perform a regression of phenotype onto proband genotype and imputed parental genotype for siblings with IBD=1. Call this $\hat{\hat{\delta}}_1$
3. The estimate of δ is then the inverse-variance weighted average of $\hat{\hat{\delta}}_0$ and $\hat{\hat{\delta}}_1$. Call this $\hat{\hat{\delta}}$.

We do not use siblings with IBD=2 since these siblings cannot provide unbiased estimates of δ. For siblings with IBD=2, the imputed parental genotype is collinear with the siblings' genotypes. For these siblings, a univariate regression of phenotype onto proband genotype estimates $\delta+((1+3F_{ST})/(1+F_{ST}))\alpha$.

We now proceed to prove that this estimator is a consistent estimator for δ, and to compute its sampling variance for the case of n independent families with two genotyped and phenotyped siblings per family, as in Section 4.2 for a random-mating population. For notational convenience, we consider that the families have been ordered by their IBD state, with families 1 to n_0 having IBD=0, families n_0+1 to n_0+n_1 having IBD=1, and families n_0+n_1+1 to n having IBD=2.

7.2.1 IBD=0

For siblings with IBD=0, the imputed parental genotype is equal to actual parental genotype, and the sum of the siblings' genotypes is equal to the actual parental genotype. The estimator is therefore both unbiased and consistent, since the imputation is unbiased (See Theorems 2 and 3). We now derive the variance of the estimator, following a procedure similar to Section 4.2.

Let $\hat{\theta}_0$ be the generalized least squares estimator. For n_0 families where the siblings are in IBD0, the variance of the generalized least-squares estimator is approximately:

$$\text{Var}(\hat{\theta}_0) \approx \left(\sum_{i=1}^{n_0} X_i^T \Sigma_i^{-1} X_i\right)^{-1}, \tag{139}$$

where

$$X_i = \begin{bmatrix} g_{i1} & g_{\text{par}(i)} \\ g_{i2} & g_{\text{par}(i)} \end{bmatrix};\ \Sigma_i^{-1} = \frac{1}{\sigma_\epsilon^2(1-r^2)} \begin{bmatrix} 1 & -r \\ -r & 1 \end{bmatrix}. \tag{140}$$

We assume that the genotypes have been mean normalised for ease of exposition. As the overall sample size tends to infinity (see (62)),

$$\sum_{i=1}^{n_0} X_i^T \Sigma_i^{-1} X_i \to \frac{n_0}{\sigma_\epsilon^2(1-r^2)} \begin{bmatrix} 2(\text{Var}(g_{i1}) - 2r\text{Cov}(g_{i1}, g_{i2}|\text{IBD=0})) & (1-r)\text{Var}(g_{\text{par}(i)}) \\ (1-r)\text{Var}(g_{\text{par}(i)}) & 2(1-r)\text{Var}(g_{\text{par}(i)}) \end{bmatrix}, \tag{141}$$

where the variances and covariances are over all families where the siblings have IBD=0. Since the IBD state of the siblings is independent of the subpopulation of the family, the proportions of families from each subpopulation in the subsample that have IBD=0 will reflect the proportions of each subpopulation in the overall sample as the sample tends to infinity.

We now compute the covariance between the siblings conditional on IBD=0:

$$\text{Cov}(g_{i1}, g_{i2}|\text{IBD=0}) = \mathbb{E}_k[\text{Cov}(g_{ki1}, g_{ki2}|\text{IBD=0})] + \text{Cov}_k(2f_k, 2f_k). \tag{142}$$

Since there is random-mating in each subpopulation and the siblings do not share any alleles IBD, $\text{Cov}(g_{ki1}, g_{ki2}|\text{IBD=0}) = 0$. Therefore,

$$\text{Cov}(g_{i1}, g_{i2}|\text{IBD=0}) = 4f(1-f)F_{ST}. \tag{143}$$

Combining this with previously derived results, we obtain

$$\sum_{i=1}^{n_0} X_i^T \Sigma_i^{-1} X_i \to \frac{4n_0 f(1-f)}{\sigma_\epsilon^2(1-r^2)} \begin{bmatrix} 1+(1-2r)F_{ST} & (1-r)(1+3F_{ST}) \\ (1-r)(1+3F_{ST}) & 2(1-r)(1+3F_{ST}). \end{bmatrix} \tag{144}$$

By inverting this matrix, we obtain the approximate large-sample variance of $\hat{\theta}_0$:

$$\text{Var}(\hat{\theta}_0) \approx \frac{\sigma_\epsilon^2}{4(1-F_{ST})(1+3F_{ST})n_0 f(1-f)} \begin{bmatrix} 2(1-r)(1+3F_{ST}) & -(1-r)(1+3F_{ST}) \\ -(1-r)(1+3F_{ST}) & 1+(1-2r)F_{ST} \end{bmatrix}. \tag{145}$$

Therefore,

$$\text{Var}(\hat{\delta}_0) \approx \frac{(1-r)\sigma_\epsilon^2}{2(1-F_{ST})n_0 f(1-f)}. \tag{146}$$

7.2.2 IBD=1

Let $\hat{\theta}_1$ be the generalized least squares estimator from siblings that have IBD=1. The generalized least-squares estimator from siblings with IBD=1 is:

$$\hat{\theta}_1 = \left(\sum_{i=n_0+1}^{n_0+n_1} X_i^T \Sigma_i^{-1} X_i \right)^{-1} \left(\sum_{i=n_0+1}^{n_0+n_1} X_i^T \Sigma_i^{-1} Y_i \right). \tag{147}$$

As the overall sample size tends to infinity,

$$\sum_{i=n_0+1}^{n_0+n_1} X_i^T \Sigma_i^{-1} X_i \rightarrow \frac{n_1}{\sigma_\epsilon^2 (1-r^2)} \begin{bmatrix} 2(\text{Var}(g_{i1}) - 2r\text{Cov}(g_{i1}, g_{i2}|\text{IBD=1})) & (1-r)\text{Cov}(\hat{g}_{\text{par}(i)}, g_{i1} + g_{i2}|\text{IBD=1}) \\ (1-r)\text{Cov}(\hat{g}_{\text{par}(i)}, g_{i1} + g_{i2}|\text{IBD=1}) & 2(1-r)\text{Var}(\hat{g}_{\text{par}(i)}|\text{IBD=1}) \end{bmatrix} \tag{148}$$

We compute the covariance between the siblings conditional on IBD=1:

$$\text{Cov}(g_{i1}, g_{i2}|\text{IBD=1}) = \mathbb{E}_k[\text{Cov}(g_{ki1}, g_{ki2}|\text{IBD=1})] + \text{Cov}_k(2f_k, 2f_k). \tag{149}$$

Since there is random-mating in each subpopulation, and the siblings share one allele IBD, $\text{Cov}(g_{ki1}, g_{ki2}|\text{IBD=1}) = f(1-f)$. Therefore,

$$\text{Cov}(g_{i1}, g_{i2}|\text{IBD=1}) = f(1-f)[1 + 3F_{ST}]. \tag{150}$$

The covariance between imputed parental genotype and the sum of siblings' genotypes conditional on IBD=1 is

$$\begin{aligned} \text{Cov}(\hat{g}_{\text{par}(i)}, g_{i1} + g_{i2}|\text{IBD=1}) &= \mathbb{E}_k[4f_k(1-f_k)] + \text{Cov}_k(3f_k + f, 4f_k) && (151) \\ &= 4f(1-f)[1 + 2F_{ST}]. && (152) \end{aligned}$$

Finally, the variance of imputed parental genotype conditional on IBD=1 is

$$\begin{aligned} \text{Var}(\hat{g}_{\text{par}(i)}|\text{IBD=1}) &= \mathbb{E}_k[3f_k(1-f_k)] + \text{Var}_k(3f_k + f) && (153) \\ &= 3f(1-f)[1 + 2F_{ST}]. && (154) \end{aligned}$$

Therefore,

$$\sum_{i=n_0+1}^{n_0+n_1} X_i^T \Sigma_i^{-1} X_i \rightarrow \frac{2n_1 f(1-f)}{\sigma_\epsilon^2 (1-r^2)} \begin{bmatrix} (2-r) + (2-3r)F_{ST} & 2(1-r)[1+2F_{ST}] \\ 2(1-r)[1+2F_{ST}] & 3(1-r)[1+2F_{ST}] \end{bmatrix}. \tag{155}$$

By inverting the above, we obtain the approximate large-sample variance of $\hat{\theta}_1$:

$$\text{Var}(\hat{\theta}_1) \approx \left(\sum_{i=n_0+1}^{n_0+n_1} X_i^T \Sigma_i^{-1} X_i \right)^{-1} \rightarrow \tag{156}$$

$$\frac{(1+r)\sigma_\epsilon^2}{2(2+r)(1-F_{ST})(1+2F_{ST})n_1 f(1-f)}\begin{bmatrix}3(1-r)(1+2F_{ST}) & -2(1-r)(1+2F_{ST})\\ -2(1-r)(1+2F_{ST}) & (2-r)+(2-3r)F_{ST}\end{bmatrix}. \tag{157}$$

To prove consistency, we also need the limit of $\sum_{i=n_0+1}^{n_0+n_1} X_i^T\Sigma_i^{-1}Y_i$:

$$\sum_{i=n_0+1}^{n_0+n_1} X_i^T\Sigma_i^{-1}Y_i \to \frac{n_1}{\sigma_\epsilon^2(1-r^2)}\begin{bmatrix}2(\text{Var}(g_{i1})-2r\text{Cov}(g_{i1},g_{i2}|\text{IBD=1})) & 2(1-r)\text{Cov}(g_{\text{par}(i)},g_{i1})\\ (1-r)\text{Cov}(\hat{g}_{\text{par}(i)},g_{i1}+g_{i2}|\text{IBD=1}) & 2(1-r)\text{Cov}(\hat{g}_{\text{par}(i)},g_{\text{par}(i)}|\text{IBD=1})\end{bmatrix}\theta \tag{158}$$

To compute the above, we need to compute $\text{Cov}(\hat{g}_{\text{par}(i)},g_{\text{par}(i)}|\text{IBD=1})$:

$$\begin{aligned}\text{Cov}(\hat{g}_{\text{par}(i)},g_{\text{par}(i)}|\text{IBD=1}) &= \mathbb{E}_k[3f_k(1-f_k)]+\text{Cov}_k(3f_k+f,4f_k) & (159)\\ &= 3f(1-f)[1+3F_{ST}]. & (160)\end{aligned}$$

Using the results derived above,

$$\sum_{i=n_0+1}^{n_0+n_1} X_i^T\Sigma_i^{-1}Y_i \to \frac{2n_1f(1-f)}{\sigma_\epsilon^2(1-r^2)}\begin{bmatrix}(2-r)+(2-3r)F_{ST} & 2(1-r)[1+3F_{ST}]\\ 2(1-r)[1+2F_{ST}] & 3(1-r)[1+3F_{ST}]\end{bmatrix}\theta. \tag{161}$$

After some algebra, this enables us to compute the limit of $\hat{\theta}_1$ as the overall sample goes to infinity:

$$\hat{\theta}_1 \to \begin{bmatrix}\delta\\ \frac{1+3F_{ST}}{1+2F_{ST}}\alpha\end{bmatrix}. \tag{162}$$

Therefore, $\hat{\delta}_1$ is a consistent estimator of δ with approximate variance

$$\text{Var}(\hat{\delta}_1)\approx\frac{3(1-r^2)\sigma_\epsilon^2}{2(2+r)(1-F_{ST})n_1f(1-f)} \tag{163}$$

7.2.3 Combining IBD=1 and IBD=0

Since both $\hat{\delta}_1$ and $\hat{\delta}_0$ are consistent estimators of δ, the inverse-variance weighted average of $\hat{\delta}_1$ and $\hat{\delta}_0$, $\hat{\delta}$, is also a consistent estimator of δ. We now compute its variance:

$$\text{Var}(\hat{\delta}) = (\text{Var}(\hat{\delta}_0)^{-1}+\text{Var}(\hat{\delta}_1)^{-1})^{-1}. \tag{164}$$

Using the above results, we have that

$$\text{Var}(\hat{\delta}_0)^{-1}+\text{Var}(\hat{\delta}_1)^{-1}\approx\frac{2(1-F_{ST})f(1-f)}{\sigma_\epsilon^2(1-r)}\left(n_0+\frac{(2+r)n_1}{3(1+r)}\right). \tag{165}$$

As the probability a sibling pair is in IBD0 is 1/4, and the probability a sibling pair is in IBD1 is 1/2, as the overall sample size, n, tends to infinity, $n_0\to n/4$ and $n_1\to n/2$. Therefore,

$$\frac{2(1-F_{ST})f(1-f)}{\sigma_\epsilon^2(1-r)}\left(n_0+\frac{(2+r)n_1}{3(1+r)}\right)\to\frac{2(1-F_{ST})f(1-f)n}{\sigma_\epsilon^2(1-r)}\left(\frac{1}{4}+\frac{(2+r)}{6(1+r)}\right). \tag{166}$$

By simplifying this expression and taking the inverse, we obtain the approximate large sample variance of $\hat{\delta}$:

$$\mathrm{Var}(\hat{\delta}) \approx \frac{6(1-r^2)\sigma_\epsilon^2}{(7+5r)(1-F_{ST})nf(1-f)}. \tag{167}$$

This can be compared with the variance of the estimator of δ from regressing phenotypic differences onto genetic differences between siblings, $\hat{\delta}_\Delta$.

$$\mathrm{Var}(\hat{\delta}_\Delta) = \frac{(1-r)\sigma_\epsilon^2}{(1-F_{ST})nf(1-f)}. \tag{168}$$

Therefore,

$$\frac{\mathrm{Var}(\hat{\delta}_\Delta)}{\mathrm{Var}(\hat{\delta})} = \frac{7+5r}{6(1+r)} = 1 + \frac{1-r}{6(1+r)}. \tag{169}$$

We therefore see that the effective sample size from this estimator is 1/6th larger than from the sibling difference estimator when $r = 0$, irrespective of F_{ST}.

7.3 Imputation from parent-offspring pairs

We consider estimating the following model:

$$Y_{ki} = \delta g_{ki} + \alpha_p g_{kp(i)} + \alpha_m g_{km(i)} + \epsilon_{ki}, \tag{170}$$

where $g_{kp(i)}$ is the genotype of the father in family i in subpopulation k, $g_{km(i)}$ is the genotype of the mother in family i in subpopulation k, and ϵ_{ki} is uncorrelated with g_{ki}, $g_{kp(i)}$, and $g_{km(i)}$.

Consider imputing the genotype of the father given the genotypes of the mother and offspring in a family:

$$\hat{g}_{kp(i)} = g_{ki}^p + f, \tag{171}$$

where g_{ki}^p is the genotype of the paternally inherited allele of the offspring in family i in subpopulation k, and $\hat{g}_{kp(i)}$ is the imputed genotype of the father. The imputation is biased since $\mathbb{E}[\hat{g}_{kp(i)}] = f_k + f \neq 2f_k$ when $f_k \neq f$.

Let Y_k be the phenotype vector in subpopulation k, and let $\hat{X}_k = [g_{ki}, \hat{g}_{kp(i)}, g_{km(i)}]$ be the corresponding matrix of observed and imputed genotypes. We examine least-squares estimation of $\theta = [\delta, \alpha_p, \alpha_m]^T$ in the overall population:

$$\hat{\theta} = (\hat{X}^T\hat{X})^{-1}\hat{X}^T Y, \text{ where } \hat{X} = \begin{bmatrix} X_1 \\ X_2 \\ \vdots \\ X_K \end{bmatrix} \text{ and } \hat{Y} = \begin{bmatrix} Y_1 \\ Y_2 \\ \vdots \\ Y_K \end{bmatrix}. \tag{172}$$

To compute the limit of $\hat{\theta}$ as the overall sample size goes to infinity, we need to compute the variances and covariances between observed and imputed genotypes in the overall population. Following the same procedure as above for imputation from sibling pairs, we obtain:

$$\mathrm{Var}(\hat{g}_{kp(i)}) = f(1-f);\ \mathrm{Cov}(g_{km(i)}, \hat{g}_{kp(i)}) = 2f(1-f)F_{ST}; \tag{173}$$

$$\mathrm{Cov}(g_{ki}, \hat{g}_{kp(i)}) = f(1-f)[1+F_{ST}];\ \mathrm{Cov}(g_{kp(i)}, \hat{g}_{kp(i)}) = f(1-f)[1+F_{ST}]. \tag{174}$$

Combining these results with the results derived for imputation from siblings, we obtain

$$\hat{\theta} \to \begin{bmatrix} 2(1+F_{ST}) & 1+F_{ST} & 1+3F_{ST} \\ 1+F_{ST} & 1 & 2F_{ST} \\ 1+3F_{ST} & 2F_{ST} & 2(1+F_{ST}) \end{bmatrix}^{-1} \begin{bmatrix} 2(1+F_{ST}) & 1+3F_{ST} & 1+3F_{ST} \\ 1+F_{ST} & 1+F_{ST} & 4F_{ST} \\ 1+3F_{ST} & 2F_{ST} & 2(1+F_{ST}) \end{bmatrix} \theta. \tag{175}$$

After some algebra, this simplifies to:

$$\hat{\theta} \to \begin{bmatrix} \delta \\ (1+c)\alpha_p \\ \alpha_m + c\alpha_p \end{bmatrix}, \text{ where } c = \frac{F_{ST}}{1+2F_{ST}}. \tag{176}$$

This shows that the estimator for the direct effects remains consistent, with biases introduced into estimates of the non-transmitted coefficients in proportion to the F_{ST} at the locus.

8 PGI analysis with assortative mating

In this section, we analyse the potential bias that could be introduced into analyses of imputed polygenic indexs for traits affected by assortative mating. In this section, we assume a simplified model for the polygenic index. We assume that there are L unlinked sites with equal weights w and equal allele frequencies f. Unlinked here means that the sites are transmitted from parents independently, so that they would be uncorrelated in a random-mating population. While this model is unrealistic, it has been used to derive many classical results on assortative mating, and applies well to polygenic indexs constructed from many genome-wide SNPs of small effect[5], as is typical for complex human traits. Let PGI_{ij} be the polygenic index of sibling j in family i, then

$$\mathrm{PGI}_{ij} = w\sum_{l=1}^{L}(g_{ijl}-2f);\ \mathrm{PGI}_{m(i)} = w\sum_{l=1}^{L}(g_{m(i)l}-2f); \mathrm{PGI}_{p(i)} = w\sum_{l=1}^{L}(g_{p(i)l}-2f); \tag{177}$$

$$\mathrm{PGI}_{\mathrm{par}(i)} = w\sum_{l=1}^{L}(g_{p(i)l}+g_{m(i)l}-4f). \tag{178}$$

where g_{ijl} is the genotype of sibling j in family i at locus l, $g_{m(i)l}$ is the genotype of the mother in family i at locus l, and $g_{p(i)l}$ is the genotype of the father in family i at locus l. Note that expectations of the PGIs are zero.

We consider imputing the parental PGI (sum of maternal and paternal PGI) from the observed genotypes of two sibling offspring of those parents at the L loci:

$$\hat{\mathrm{PGI}}_{\mathrm{par}(i)} = w\sum_{l=1}^{L}(\hat{g}_{\mathrm{par}(i)l}-4f), \tag{179}$$

where

$$\hat{g}_{\text{par}(i)l} = \begin{cases} g_{i1l} + g_{i2l} = g_{\text{par}(i)l}, & \text{if } \text{IBD}_l = 0 \\ g_{i1l} + g_{i2l}^k + f, & \text{if } \text{IBD}_l = 1 \\ g_{i1l} + 2f, & \text{if } \text{IBD}_l = 2, \end{cases} \tag{180}$$

where g_{i2}^k is the allele in sibling 2 that is not shared IBD with the alleles of sibling 1. We note that $\hat{g}_{\text{par}(i)} = \mathbb{E}[g_{\text{par}(i)}|g_{i1}, g_{i2}, \text{IBD}]$ under random-mating, but is not equal to this when there is assortative mating due to correlations between alleles transmitted to the siblings and those not transmitted to the siblings. Under random-mating, we have that

$$\hat{\text{PGI}}_{\text{par}(i)} = \mathbb{E}[\text{PGI}_{\text{par}(i)}|\{g_{i1l}, g_{i2l}, \text{IBD}_l\}_{l=1}^L]; \tag{181}$$

i.e. the imputed parental PGI is the conditional expectation of the parental PGI given the genotypes of the siblings at the L loci and the IBD state of the siblings at those loci. However, this is not the case under assortative mating.

We consider assortative mating that has reached an equilibrium where

$$r_{am} = \text{Corr}(\text{PGI}_{p(i)}, \text{PGI}_{m(i)}), \tag{182}$$

and therefore the correlations between distinct alleles in the parents and offspring are all equal to

$$m = \frac{r_{am}}{2L(1 - r_{am}) + r_{am}}, \tag{183}$$

as given first by Wright[5, 6].

8.1 Joint distribution of observed and imputed PGIs

We derive the equilibrium variances and covariances between parental, imputed parental, and offspring PGIs in Appendix A. Let $\mathbf{Z}_{ij} = [\text{PGI}_{ij},\ \hat{\text{PGI}}_{\text{par}(i)},\ \text{PGI}_{\text{par}(i)}]^T$, and let V_0 be the variance of the PGI in a random mating population. As $L \to \infty$,

$$\text{Cov}(\mathbf{Z}_{ij}) \to \frac{V_0}{1 - r_{am}} \begin{bmatrix} 1 & 1 + r_{am}/2 & 1 + r_{am} \\ 1 + r_{am}/2 & (3/2)(1 + r_{am}/2) & (3/2)(1 + r_{am}) \\ 1 + r_{am} & (3/2)(1 + r_{am}) & 2(1 + r_{am}) \end{bmatrix}. \tag{184}$$

8.2 Estimating the direct effect of a PGI

We consider estimating $\theta = [\delta, \alpha]^T$ from the model

$$Y_{ij} = \delta \text{PGI}_{ij} + \alpha \text{PGI}_{\text{par}(i)} + \epsilon_{ij}, \tag{185}$$

by least-squares regression on $[\text{PGI}_{ij},\ \hat{\text{PGI}}_{\text{par}(i)}]$. Let $\hat{\theta}$ be the resulting estimator of θ, then, given that ϵ_{ij} is uncorrelated with PGI_{ij} and $\text{PGI}_{\text{par}(i)}$,

$$\hat{\theta} \to \begin{bmatrix} 1 & 1+r_{am}/2 \\ 1+r_{am}/2 & (3/2)(1+r_{am}/2) \end{bmatrix}^{-1} \begin{bmatrix} 1 & 1+r_{am} \\ 1+r_{am}/2 & (3/2)(1+r_{am}) \end{bmatrix} \theta \tag{186}$$

$$= \frac{1}{1-r_{am}} \begin{bmatrix} 3 & -2 \\ -2 & \frac{4}{2+r_{am}} \end{bmatrix} \begin{bmatrix} 1 & 1+r_{am} \\ 1+r_{am}/2 & (3/2)(1+r_{am}) \end{bmatrix} = \begin{bmatrix} \delta \\ \frac{2(1+r_{am})}{2+r_{am}}\alpha \end{bmatrix}. \tag{187}$$

We therefore see that the bias in estimation of α is equal to $\frac{r_{am}}{2+r_{am}}\alpha$, and that estimation of δ is consistent. Univariate regression of proband phenotype onto proband PGI gives an expected coefficient of $\delta + (1 + r_{am})\alpha$, and subtracting δ from this results in an estimate of α that is more biased: $(1 + r_{am})\alpha$.

In general, the imputed parental PGIs do not capture fully the inflation of variance due to assortative mating, so that the imputation is biased by a multiplicative factor related to the strength of assortative mating, leading to a multiplicative bias in the estimates of non-transmitted coefficients, but not the direct or indirect sibling effect estimates.

8.3 Adjusting for the bias introduced by assortative mating

For the analysis of the educational attainment PGI in the main text, we used Equation 187 to adjust for the bias in α introduced by assortative mating. In this analysis, parental genotypes were imputed from sibling genotypes (without observed parental genotypes) in 88.8% of the sample, and for 92.8% of those individuals, the parental genotypes were imputed from a single sibling pair. This suggests that Equation 187 should provide a good approximation, provided that AM has reached an approximate equilibrium. The formula implies that parental effect estimates are inflated by a factor of $(1+r_{am})/(1+r_{am}/2)$, where r_{am} is the correlation between the maternal and paternal PGI at equilibrium. To compute r_{am}, we took advantage of the fact that the correlation between siblings' PGI values is equal to $(1 + r_{am})/2$ at equilibrium (see Nagylaki[7]). We estimated the correlation between siblings' PGI to be 0.557, giving an estimate of 0.114 for r_{am}, implying that average NTC estimates are inflated by a factor of around 1.054. We therefore divided the average NTC estimates by 1.054 to produce adjusted NTC estimates (Supplementary Tables 3 and 5).

9 Inferring IBD between siblings

We infer the identity-by-descent sharing states of a sibling pair by using a Hidden Markov Model (HMM). Let g_{ijl} be the un-phased genotype (0, 1, or 2) of sibling j in family i at SNP $l = 1, \dots, L$, and let $\text{IBD}_{il} \in \{0, 1, 2\}$ be the IBD state of the sibling pair at SNP l. We consider that SNPs $l = 1, \dots, L$ are ordered by position on a single chromosome, and that the genetic map is known, so that $d(l, l')$ gives the distance between SNPs l and l' in centiMorgans

(cM). The goal of inference is to find the path, $\text{IBD}_i = \{\text{IBD}_{il}\}_{l=1}^{L}$, that maximises the joint probability

$$\mathbb{P}(\text{IBD}_i, \{g_{i1l}\}_{l=1}^{L}, \{g_{i2l}\}_{l=1}^{L}). \tag{188}$$

In order to make this problem tractable, we make some simplifying assumptions. First, we assume that the IBD states follow a Markov process such that

$$\mathbb{P}(\text{IBD}_{i(l+1)}|\{\text{IBD}_{ik}\}_{k=1}^{l}) = \mathbb{P}(\text{IBD}_{i(l+1)}|\text{IBD}_{il}). \tag{189}$$

Note that there are four independent meioses leading to the two siblings' genotypes: one for each parent for each sibling. To compute $\mathbb{P}(\text{IBD}_{i(l+1)}|\text{IBD}_{il})$, we consider the probability that there is a recombination event during one of the meioses given a distance of d cM:

$$\mathbb{P}(\text{recombination} \,|\, d\,\text{cM}) = \mathbb{P}(\text{Odd number of cross-overs in d cM}) = \frac{1-\exp(-d/50)}{2}. \tag{190}$$

Therefore, the probability of at least one recombination across the four independent meioses given a distance of d cM is:

$$\mathbb{P}(\text{at least 1 recombination in two sibs} \,|\, d\,\text{cM}) = 1 - \left(\frac{1+\exp(-d/50)}{2}\right)^4 = \rho(d). \tag{191}$$

We make the simplifying assumption that the IBD state changes only by 1 between subsequent SNPs. By symmetry, a transition from IBD=1 to IBD=0 is of equal probability to a transition from IBD=1 to IBD=2; therefore, the transition matrix between SNPs l and $l+1$ with distance d cM is:

$$\begin{array}{c} \\ 0 \\ 1 \\ 2 \end{array}\begin{array}{c} \begin{array}{ccc} 0 & 1 & 2 \end{array} \\ \begin{pmatrix} 1-\rho(d) & \rho(d) & 0 \\ \rho(d)/2 & 1-\rho(d) & \rho(d)/2 \\ 0 & \rho(d) & 1-\rho(d) \end{pmatrix} \end{array}. \tag{192}$$

Since SNPs on the same chromosome tend to be in linkage disequilibrium, probabilities of observing sibling genotypes at state l and state $l+1$ are not independent. Working with the full joint-distribution of sibling genotypes and IBD states would make inference computationally challenging. We instead approximate the full joint-distribution by weighting the contribution to the log-likelihood from the siblings' genotypes at each SNP according to the inverse of the SNP's LD-score, as in Speed et al.[8] for the problem of inferring variance components from SNP-level summary statistics. Let λ_l be the LD-score of SNP l. We can thereby compute the approximate joint log-likelihood as

$$\log(\mathbb{P}(\text{IBD}_i, \{g_{i1l}\}_{l=1}^{L}, \{g_{i2l}\}_{l=1}^{L})) \approx \tag{193}$$

$$\log(\mathbb{P}(\text{IBD}_{i1})) + \sum_{l=2}^{L} \log(\mathbb{P}(\text{IBD}_{il}|\text{IBD}_{i(l-1)})) + \sum_{l=1}^{L} \lambda_l^{-1} \log(\mathbb{P}(g_{i1l}, g_{i2l}|\text{IBD}_{il})). \tag{194}$$

The probabilities of the initial states are given by the Laws of Mendelian Inheritance, with $\mathbb{P}(\text{IBD}_{i1} = 2) = \mathbb{P}(\text{IBD}_{i1} = 0) = 0.25$. Let f_l be the allele frequency of the allele being counted at SNP l, with $\mathbb{E}[g_{ijl}] = 2f_l$. The probabilities of the siblings' genotypes given IBD state are given assuming random-mating:

$$\mathbb{P}(g_{i1l}, g_{i2l}|\text{IBD}_{i1} = 0) = \mathbb{P}(g_{i1l})\mathbb{P}(g_{i2l}), \tag{195}$$

where $g_{ijl} \sim \text{Binomial}(2, f_l)$. For IBD=2, $\mathbb{P}(g_{i1l}, g_{i2l}|\text{IBD}_{i1} = 2) = \mathbb{P}(g_{i1l})$ if $g_{i1l} = g_{i2l}$; and $\mathbb{P}(g_{i1l}, g_{i2l}|\text{IBD}_{i1} = 2) = 0$, if $g_{i1l} \neq g_{i2l}$. For IBD=1, the joint probabilities are given in Supplementary Note Table 5.

The above defines a Hidden Markov Model with hidden states IBD=0, IBD=1, IBD=2. The path that maximises (194), $\hat{\text{IBD}}_i$, can be computed in $O(L)$ operations using the Viterbi algorithm[9].

9.1 With genotyping errors

In the above, we assumed that genotypes are observed without error. In applications to real data, genotyping errors occur typically at a very low rate. Even at a low rate, genotyping errors can cause problems for inferring IBD segments. Consider a genotyping error in an IBD=2 region leading to a difference between the siblings' genotypes: this is not possible according to a model without genotyping errors, so the algorithm would be forced to transition in and out of IBD=1 to accommodate the genotyping error.

We introduce an additional layer to the above model that allows for observed genotypes to differ from true genotypes due to genotyping error. We assume that the observed genotype can only differ from the true genotype by 1[10]. Let $\tilde{g}_{ijl}$ be the observed genotype of sibling j in family i at SNP l. Given a genotyping error probability of γ, we model errors as

$$\mathbb{P}(\tilde{g}_{ijl}|g_{ijl}) = 1 - \gamma, \text{ if } \tilde{g}_{ijl} = g_{ijl}; \tag{196}$$

$$\mathbb{P}(\tilde{g}_{ijl}|g_{ijl} = 1) = \gamma/2, \text{ if } \tilde{g}_{ijl} \neq g_{ijl}; \tag{197}$$

$$\mathbb{P}(\tilde{g}_{ijl}|g_{ijl} \neq 1) = \gamma, \text{ if } |\tilde{g}_{ijl} - g_{ijl}| = 1; \tag{198}$$

$$\mathbb{P}(\tilde{g}_{ijl}|g_{ijl} \neq 1) = 0, \text{ if } |\tilde{g}_{ijl} - g_{ijl}| = 2. \tag{199}$$

We assume that genotyping errors are independent between siblings and independent of the IBD state and l. The probability of the observed genotypes given the IBD state is therefore:

$$\mathbb{P}(\tilde{g}_{i1l}, \tilde{g}_{i2l}|\text{IBD}_{il}) = \sum_{g_{i1l}, g_{i2l}} \mathbb{P}(\tilde{g}_{i1l}|g_{i1l})\mathbb{P}(\tilde{g}_{i2l}|g_{i2l})\mathbb{P}(g_{i1l}, g_{i2l}|\text{IBD}_{il}). \tag{200}$$

We find the path, $\hat{\text{IBD}}_i$, that maximises

$$\log(\mathbb{P}(\text{IBD}_{i1})) + \sum_{l=2}^{L} \log(\mathbb{P}(\text{IBD}_{il}|\text{IBD}_{i(l-1)})) + \sum_{l=1}^{L} \lambda_l^{-1} \log(\mathbb{P}(\tilde{g}_{i1l}, \tilde{g}_{i2l}|\text{IBD}_{il})). \tag{201}$$

9.2 Smoothing

Even with a genotyping error model, we found that the above model tended to produce some very short IBD segments indicative of overfitting (possibly due to high levels of local LD) or excessive genotyping errors in an individual or a SNP not well captured by the genotyping error model. We therefore added a simple routine to smooth the Viterbi path. If an IBD segment differs in state from its adjacent segments, and the adjacent segments have the same state as each other, and the IBD segment's length in cM is below a threshold m, we set the IBD state of the SNPs covered by that segment to be equal to the IBD state of the adjacent segments.

9.3 Parameter optimization

For the application to UKB data, in order to estimate the accuracy of the inferred IBD segments and to choose optimal parameters γ and m, we used 31 families where two siblings and both of their parents have been genotyped (quads) to infer the true IBD state for a subset of SNPs: when both parents are heterozygous, the IBD state of the siblings is equal to 2 minus the absolute difference in the siblings' genotypes, except when both siblings are heterozygous. We smoothed the true IBD inferred from the quads to account for genotyping errors: if the IBD state at a SNP differed from its neighbours, and both neighbours had the same IBD state, we changed the IBD state of the SNP to be the same as its neighbours.

To infer the IBD segments between siblings, we used the un-phased SNPs on the UKB genotyping array with MAF>1%. We chose the parameters γ and m by performing a grid search over $log_{10}(\gamma)$ from -5 to -1 in increments of 0.5, and for $log_{10}(P_{cM}) = log_{10}(1 - \exp(-m/100))$ from -5 to -1 in increments of 0.5. P_{cM} is the probability of observing a segment as short or shorter than m. For each tuple (γ, m), we calculated the probability of inferring the correct IBD state by comparing the inferred IBD state to the true IBD state for all SNPs where we could infer the true IBD state. We found that $(\gamma, m) = (10^{-4}, 0.01\,\text{cM})$ gave the highest probability of inferring the true IBD state, 99.65%. We give the proportions of SNPs with inferred IBD states 0, 1, and 2 as a function of the true IBD state in Supplementary Table 1.

10 Mixed model inference

We use a mixed model to account for correlations between individuals within a family. The data are comprised of observations on n families. For family i, there are n_i observations, giving a total of $\sum_{i=1}^{n} n_i = N$ observations. We assume that the data has been ordered so that the observations from family 1 are indexed from 1 to n_1, the observations from individual 2 are indexed from $n_1 + 1$ to $n_1 + n_2$, etc.

The phenotype is an $[N \times 1]$ vector Y and the covariate matrix is an $[N \times c]$ matrix X. The X matrix can be constructed using the genotypes of the siblings and/or (imputed) parents in different ways depending on the application. We introduce a $[N \times n]$ matrix Z such that $[Z]_{ij} = 1$ if observation i is from family j, and $[Z]_{ij}$ is zero otherwise. We assume that the

within-family means are independently normally distributed, represented by an $[n \times 1]$ vector $u \sim \mathcal{N}(0, \sigma_F^2 \mathrm{I}_n)$. The model is

$$Y = X\theta + Zu + \epsilon. \tag{202}$$

We assume that the residuals are I.I.D. Gaussians, $\epsilon \sim N(0, \sigma^2 I)$. The distribution of $Y|X$ is therefore,

$$Y|X \sim \mathcal{N}(X\theta, \sigma_F^2 ZZ^T + \sigma_\epsilon^2 I). \tag{203}$$

It can readily be inferred that ZZ^T has a simple block-diagonal structure. For $n = 2$, the matrix has the following structure:

$$ZZ^T = \begin{bmatrix} 1_{n_1}1_{n_1}^T & 0 \\ 0 & 1_{n_2}1_{n_2}^T \end{bmatrix}, \tag{204}$$

where 1_k is the $[k \times 1]$ column vector of all 1s.

10.1 Loss function and gradients

Instead of the optimising the likelihood, we seek to minimise negative two times the log-likelihood as a loss function:

$$L = \log|\Sigma| + (y - X\theta)^T \Sigma^{-1} (y - X\theta), \tag{205}$$

where $\Sigma = \sigma_F^2 ZZ^T + \sigma_\epsilon^2 I$.

Naive computation of the loss function takes $\mathrm{O}(N^3)$ operations. However, the likelihood component of the loss function can be split into a sum over families. Let Σ_i be the diagonal block of Σ corresponding to observations on family i. Furthermore, let y_i be the $[n_i \times 1]$ vector of observations for individual i, and let X_i be the $[n_i \times c]$ matrix of covariate observations. Then,

$$L = \sum_{i=1}^{n} \log|\Sigma_i| + \sum_{i=1}^{n} (y_i - X_i\theta)^T \Sigma_i^{-1} (y_i - X_i\theta), \tag{206}$$

It is straightforward to show that the MLE for θ, $\hat{\theta}$, given the variance parameters, corresponds to the generalised least-squares estimator:

$$\hat{\theta} = \left(\sum_{i=1}^{n} X_i^T \Sigma_i^{-1} X_i \right)^{-1} \left(\sum_{i=1}^{n} X_i^T \Sigma_i^{-1} y_i \right). \tag{207}$$

It is also straightforward to show that the asymptotic sampling variance of the MLE for θ is:

$$\mathrm{Var}(\hat{\theta}) = \left(\sum_{i=1}^{n} X_i^T \Sigma_i^{-1} X_i \right)^{-1}. \tag{208}$$

To derive the gradient with respect to the variance parameters, we introduce $\tau = \sigma_\epsilon^2/\sigma_F^2$, and parameterise the model in terms of τ and σ_ϵ^2. Because the blocks of Σ are comprised of a diagonal plus a rank-one matrix, the determinant and inverse of each block can be computed analytically using the Sherman-Morrison-Woodbury identity and the Matrix Determinant Lemma. This gives

$$\Sigma_i^{-1} = \frac{1}{\sigma_\epsilon^2}\left(\mathrm{I}_{n_i} - \frac{1_{n_i}1_{n_i}^T}{\tau + n_i}\right);\ \log|\Sigma_i| = n_i \log(\sigma_\epsilon^2) + \log\left(1 + \frac{n_i}{\tau}\right). \tag{209}$$

The loss function can thus be expressed as

$$L = N\log(\sigma_\epsilon^2) + \sum_{i=1}^{n} \log\left(1 + \frac{n_i}{\tau}\right) + \frac{(y - X\theta)^T(y - X\theta)}{\sigma_\epsilon^2} - \frac{1}{\sigma_\epsilon^2}\sum_{i=1}^{n}\frac{[1_{n_i}^T(y_i - X_i\theta)]^2}{\tau + n_i}, \tag{210}$$

which can be computed in $\mathrm{O}(N)$ operations. From this, we derive expressions for the gradient with respect to the variance parameters that can also be computed in $\mathrm{O}(N)$ operations:

$$\frac{\partial L}{\partial \sigma_\epsilon^2} = \frac{N}{\sigma_\epsilon^2} - \frac{(y - X\theta)^T(y - X\theta)}{\sigma_\epsilon^4} + \frac{1}{\sigma_\epsilon^4}\sum_{i=1}^{n}\frac{[1_{n_i}^T(y_i - X_i\theta)]^2}{\tau + n_i}; \tag{211}$$

and

$$\frac{\partial L}{\partial \tau} = \frac{1}{\sigma_\epsilon^2}\sum_{i=1}^{n}\frac{[1_{n_i}^T(y_i - X_i\theta)]^2}{(\tau + n_i)^2} - \sum_{i=1}^{n}\frac{n_i}{\tau(\tau + n_i)}. \tag{212}$$

10.2 Optimisation

The parameters we are optimising over are $\theta = (\theta, \sigma_\epsilon^2, \tau)$. However, since the MLE for θ can be computed efficiently analytically given an estimate of τ, we instead optimise

$$L_{\text{prof}}(\sigma_\epsilon^2, \tau) = L(\hat{\theta}(\tau), \sigma_\epsilon^2, \tau), \tag{213}$$

where the optimisation takes place over $(\sigma_\epsilon^2, \tau)$ only, with the MLE for θ for a given τ, $\hat{\theta}(\tau)$, computed analytically.

We optimise the model with the L-BFGS-B algorithm, with $(\sigma_\epsilon^2, \tau)$ bounded below at $(10^{-5}, 10^{-5})$. For application to a set of SNPs from a chromosome, a null model including no SNPs is first fit. By default, we initialise $(\sigma_\epsilon^2, \tau)$ to $(s_Y^2/2, 1)$, where s_Y^2 is the sample estimate of the phenotypic variance. The MLEs of τ and σ_ϵ^2 from the null model are then fixed for all SNP specific models, allowing analytical computation of the (approximate) MLE for θ for each SNP. To do this, we first transform the phenotype vector and X by the inverse square root of Σ given the MLEs for $(\sigma_\epsilon^2, \tau)$, which can be computed efficiently given the block-diagonal structure of Σ. Given this transformation, computation of the MLE for α reduces to ordinary least squares (OLS). This can be done once for all SNPs reducing the problem for multiple SNPs to repeated OLS.

Estimating effects for N individuals and M SNPs therefore takes $\mathrm{O}(N)$ operations to fit the variance parameters, and $\mathrm{O}(NM)$ operations to transform the phenotype and genotypes and estimate SNP effects, given the transformed data, through repeated OLS.

11 Estimating genome-wide correlations between effects

To estimate correlations between different types of effect, such as direct effects and population effects, we give here a moment-based estimator that accounts for the sampling errors in the effect estimates. For example, let $\hat{\delta}_l$ be the direct effect estimate for SNP l, and let $\hat{\beta}_l$ be the estimated population effect. Then we have that

$$\hat{\delta}_l = \delta_l + \epsilon_{\delta l}; \ \hat{\beta}_l = \beta_l + \epsilon_{\beta l}; \tag{214}$$

where δ_l is the true direct effect for that SNP, and $\epsilon_{\delta l}$ is the sampling error; and β_l is the true population effect for that SNP, and $\epsilon_{\beta l}$ is the sampling error. We assume that the variance-covariance matrix of the sampling errors at each SNP is known:

$$\text{Var}\left(\begin{bmatrix}\hat{\delta}_l \\ \hat{\beta}_l\end{bmatrix}\right) = \begin{bmatrix}\sigma^2_{\delta l} & r_{\delta\beta l}\sigma_{\delta l}\sigma_{\beta l} \\ r_{\delta\beta l}\sigma_{\delta l}\sigma_{\beta l} & \sigma^2_{\beta l}\end{bmatrix}, \tag{215}$$

where $\sigma^2_{\delta l}$ is the sampling variance for the direct effect of SNP l, $\sigma^2_{\beta l}$ is the sampling variance for the population effect of SNP l, and $r_{\delta\beta l}$ is the sampling correlation between the direct and population effects for SNP l.

We aim to estimate the genome-wide correlation between the true effects:

$$r(\delta, \beta) = \frac{\text{Cov}(\delta_l, \beta_l)}{\sqrt{\text{Var}(\delta_l)\text{Var}(\beta_l)}}. \tag{216}$$

Assuming that the true effects have expectation zero across the SNPs and by applying the Law of Total Variance, we can express the correlation between the true effects as:

$$r(\delta, \beta) = \frac{\text{Cov}(\hat{\delta}_l, \hat{\beta}_l) - \mathbb{E}[\text{Cov}(\epsilon_{\delta l}, \epsilon_{\beta l})]}{\sqrt{(\text{Var}(\hat{\delta}_l) - \mathbb{E}[\text{Var}(\epsilon_{\delta l})])(\text{Var}(\hat{\beta}_l) - \mathbb{E}[\text{Var}(\epsilon_{\beta l})]))}} \tag{217}$$

We use weighted sample estimates of these quantities to obtain our estimator:

$$\hat{r}(\delta, \beta) = \frac{\sum_l w_l(\hat{\delta}_l\hat{\beta}_l - r_{\delta\beta l}\sigma_{\delta l}\sigma_{\beta l})}{\sqrt{(\sum_l w_l(\hat{\delta}^2_l - \sigma^2_{\delta l}))(\sum_l w_l(\hat{\beta}^2_l - \sigma^2_{\beta l}))}}. \tag{218}$$

Similar to LDSC[11], we use $w_l = (f_l(1 - f_l))/\lambda_l$ as the weight for SNP l, where f_l is the allele frequency, and λ_l is the LD-score of SNP l. The weighting in proportion to $f_l(1 - f_l)$ approximately equalizes the sampling variances across SNPs, and the weighting in proportion to λ_l^{-1} accounts for correlations between SNPs due to local LD.

For the analyses in the main text, we used the LDSC software package with a 1cM window to compute LD scores. To estimate standard errors, we used the same block-jacknife approach as LDSC with 200 blocks. We excluded SNPs with MAF<5%. We used the sampling correlation

between direct and population effects as a further form of quality control. Outlying values of this correlation are indicative of IBD inference errors or low genotyping quality. We excluded SNPs where, for any trait, the inferred sampling correlation between direct and population effects differed by more than 6 standard deviations from the mean across all SNPs, excluding 101 SNPs.

12 Simulations

12.1 Artificial populations

We simulated 1,000 SNPs for 3,000 parent-pairs. We simulated phased parental genotypes by drawing from a Bernoulli(0.5) distribution for the presence/absence of the allele on each haplotype. For each parent-pair, we generated two full-sibling offspring. We generated phased and unphased offspring genotypes and IBD segments by simulating meiosis without recombination. For the comparison to AlphaFamImpute, we set different sets of genotypes to missing to test different imputation scenarios, and we imputed missing parental genotypes using *snipar* and AlphaFamImpute.

To check the theoretical results on sampling variance, we simulated phenotypes for the offspring by drawing direct, paternal, and maternal effects for each SNP from a multivariate normal distribution with correlations of 0.5 between the different effects. We scaled the resulting effects so that the combined phenotypic variance explained by direct, paternal, and maternal effects was equal to a given value. We simulated three phenotypes where the combined variance explained by direct, paternal, and maternal effects was 0.4, 0.2, and 0, generating correlations between siblings' phenotypes of 0.336, 0.160, and 0, respectively.

We set all parents' genotypes to missing, and we imputed the parental genotypes using *snipar* applied to both phased and unphased sibling genotypes. We estimated direct, paternal, and maternal effects using *snipar*. To compare results to not performing imputation, we estimated direct effects by regression of proband phenotype onto one half of the difference between the proband's genotype and the proband's sibling's genotype within the same mixed model framework. We compared the average sampling variance of direct effect estimates when using parental genotypes imputed from both phased and unphased data to the average sampling variance of direct effect estimates from differences between sibling genotypes (Extended Data Figure 3).

12.2 UK Biobank simulations

We simulated multiple realistic populations based on phased haplotypes at 146,634 autosomal HapMap3 SNPs present on the UKB genotyping array. We used 100,000 randomly selected, unrelated individuals from the 'White British' subsample of the UKB. We paired individuals into parent-pairs based on random-mating, assortative mating, or by UKB assessment center, depending on the phenotype. We simulated two offspring for each parent-pair by simulating

meiosis in each parent using a European recombination map distributed as part of the Eagle software package[12]; we recorded the IBD segments shared between siblings based upon the simulated recombination events. We set parental genotypes as missing and imputed them from unphased genotypes using *snipar*, and we inferred direct effects and NTCs using the linear mixed model in *snipar*, as outlined in Section 10.

For the phenotypes without AM or population stratification, we randomly mated the 100,000 individuals to create 50,000 mother-father pairs that produced 50,000 sibling pairs as offspring. We simulated phenotypes in the offspring generation by choosing 1,500 SNPs at random to be causal. For each SNP, we simulated direct effects and parental indirect genetic effects (IGEs) — chosen to be equal between paternal and maternal — from a bivariate normal distribution with equal variances and different levels of correlation: 0, 0.5, and 1. We scaled the resulting effects so that the total variance explained by combined direct effects and parental IGEs was 75% of the phenotypic variance, with the remaining phenotypic variance due to Gaussian noise. We also simulated a trait without parental IGEs where the direct effects explained 75% of the phenotypic variance.

12.2.1 Simulating assortative mating

We simulated a separate population with a phenotype affected by direct genetic effects and AM. We simulated the first generation by randomly pairing individuals into 50,000 parent pairs, and we simulated offspring by simulating meiosis as above. We simulated direct effects for 1,500 randomly selected SNPs and scaled the effects so that the variance explained by the direct effects was 50% in the first offspring generation generated by random-mating. We then simulated subsequent generations by pairing parents according to their ranks in an ordered list, where the ordering was determined by the value of their phenotype plus a Gaussian noise term.

Specifically, let Y_{ij} be the phenotype of the sibling j in family i. Let sibling 1 be the male sibling and sibling 2 be the female sibling for all families. We ordered the males by $Z_{i1} = Y_{i1} + u_{i1}$, and we ordered the females by $Z_{i2} = Y_{i2} + u_{i2}$, where the u_{ij} are independent and identically distributed as $\mathcal{N}(0, (1/r_y - 1)\text{Var}(Y))$, where r_y is the desired phenotypic correlation between parents, and $\text{Var}(Y)$ is the phenotypic variance in that generation. We chose $r_y = 0.5$, and we iterated this procedure for 20 generations, reaching an approximate equilibrium: the relative increase in phenotypic variance from the penultimate to final generation was 0.036%.

The heritability in the final generation was 58.20%, and the variance due to direct effects was 40.82% higher than in the first generation (generated by random-mating). Theory implies that the genetic variance at equilibrium should be a factor of $1/(1 - h^2_\infty r_y)$ larger than the genetic variance in a random-mating population, where h^2_∞ is the equilibrium heritability and r_y is the phenotypic correlation of parents[7]. Here, $h^2_\infty = 0.5820$ and $r_y = 0.5$, giving a theoretical prediction that the genetic variance should be inflated by a factor of $1/(1-0.5820*0.5) = 1.4104$ at equilibrium, close to the 40.8% increase we observed in our simulation.

12.2.2 Simulating vertical transmission

A simple model for vertical transmission[13] is that the phenotype of the offspring is affected by the same phenotype in the parents:

$$Y_{ij} = \sum_{l=1}^{L} \delta_l g_{ijl} + b_p Y_{p(i)} + b_m Y_{m(i)} + \epsilon_{ij}, \tag{219}$$

where g_{ijl} is the genotype of sibling j in family i at SNP l, δ_l is the direct effect of SNP l, $Y_{p(i)}$ is the phenotype of the father in family i, $Y_{m(i)}$ is the phenotype of the mother, and b_p and b_m are coefficients that determine the strength and direction of vertical transmission from fathers and mothers respectively. The phenotypes of the mother and father also depend on the phenotypes of their parents, and so on. The phenotype distribution under vertical transmission reaches an equilibrium provided that b_p and b_m are not too large[13].

To simulate a phenotype affected by vertical transmission at equilibrium, we first simulated direct genetic effects from a Gaussian distribution for 1,500 randomly selected SNPs, and we scaled the effects so that the heritability (without vertical transmission) was 50%. We simulated the first offspring generation by randomly pairing individuals in the first generation into 50,000 parent pairs and simulated offspring by simulating meiosis as above. We simulated the phenotypes of the first offspring generation using the following formula:

$$Y_{ij} = \sum_{l=1}^{L} \delta_l g_{ijl} + (1/4)(Y_{p(i)} + Y_{m(i)}) + \epsilon_{ij}, \tag{220}$$

where the direct effects were the same as in the base generation. We iterated this process for 20 generations, reaching an approximate equilibrium where the heritability was 32.7%. To simulate vertical transmission with assortative mating[14], we followed the same procedure, except we followed the procedure outlined above for the phenotype with assortative mating to create parent pairs whose phenotypic correlation was 0.5. The heritability in the base generation was 50% and declined to 29% in the final generation even though the variance due to direct genetic effects increased by 59%.

12.2.3 Simulating population stratification

We simulated a separate population with a phenotype influenced by direct genetic effects and population stratification. To simulate population structure that reflects the geographic structure in the UKB sample, we divided the sample up by the center they were assessed at (19 centers in total), and we randomly paired individuals into parent-pairs within each assessment center, generating two full sibling offspring for each parent-pair as above. We then combined the simulated data from all of the centers. To simulate a phenotype affected by population stratification, we simulated direct effects for 1,500 randomly selected SNPs, scaled so that they explained 50% of the phenotypic variance, and we simulated normally distributed 'center effects'

for each center, so that the mean phenotype differed between assessment centers. The 'center effects' were scaled so that they explained 30% of the phenotypic variance in the combined sample, with the remaining 20% of phenotypic variance due to Gaussian noise.

References

[1] Young, A. I., Benonisdottir, S., Przeworski, M., and Kong, A. Deconstructing the sources of genotype-phenotype associations in humans. *Science*, **365**(6460):1396–1400 2019.

[2] Kong, A., et al. The nature of nurture: Effects of parental genotypes. *Science*, **359**(6374):424–428 2018. ISSN 10959203. doi:10.1126/science.aan6877.

[3] Young, A. I., et al. Relatedness disequilibrium regression estimates heritability without environmental bias. *Nature Genetics*, **50**(9):1304–1310 2018. ISSN 1546-1718. doi:10.1038/s41588-018-0178-9.

[4] Hwang, L.-D., et al. Estimating indirect parental genetic effects on offspring phenotypes using virtual parental genotypes derived from sibling and half sibling pairs. *PLoS genetics*, **16** 2020.

[5] Crow, J. F. and Kimura, M. *An introduction to population genetics theory.* New York, Evanston and London: Harper & Row, Publishers 1970.

[6] Wright, S. Systems of mating. III. Assortative mating based on somatic resemblance. *Genetics*, **6**(2):144 1921.

[7] Nagylaki, T. Assortative mating for a quantitative character. *Journal of Mathematical Biology*, **16**(1):57–74 1982. ISSN 14321416. doi:10.1007/BF00275161.

[8] Speed, D., Holmes, J., and Balding, D. J. Evaluating and improving heritability models using summary statistics. *Nature Genetics*, **52**(4):458–462 2020.

[9] Durbin, R., Eddy, S. R., Krogh, A., and Mitchison, G. *Biological sequence analysis: probabilistic models of proteins and nucleic acids.* Cambridge university press 1998.

[10] Saunders, I. W., Brohede, J., and Hannan, G. N. Estimating genotyping error rates from Mendelian errors in SNP array genotypes and their impact on inference. *Genomics*, **90**(3):291–296 2007.

[11] Bulik-Sullivan, B., et al. An atlas of genetic correlations across human diseases and traits. *Nature Genetics*, **47**(11):1236–1241 2015. ISSN 15461718. doi:10.1038/ng.3406.

[12] Loh, P. R., Palamara, P. F., and Price, A. L. Fast and accurate long-range phasing in a UK Biobank cohort. *Nature Genetics*, **48**(7):811–816 2016. ISSN 15461718. doi:10.1038/ng.3571.

[13] Cavalli-Sforza, L. L. and Feldman, M. W. Cultural versus biological inheritance: Phenotypic transmission from parents to children (A theory of the effect of parental phenotypes on children's phenotypes). *American journal of human genetics*, **25**:618–637 1973. ISSN 0002-9297.

[14] Rice, J., Cloninger, C. R., and Reich, T. Multifactorial inheritance with cultural transmission and assortative mating. I. Description and basic properties of the unitary models. *American journal of human genetics*, **30**(6):618–643 1978. ISSN 0002-9297.

A Equilibrium distribution of observed and imputed PGIs

A.1 Equilibrium variance of PGI

We compute the variance of the PGI at equilibrium in terms of r and the variance of the PGI in a random mating population, V_0:

$$V_0 = Lw^2 2f(1-f). \tag{221}$$

The variance at equilibrium is:

$$\begin{aligned}
\text{Var}(\text{PGI}_{ij}) &= w^2\left(\sum_{l=1}^{L}\text{Var}(g_{ijl}) + \sum_{l\neq k}^{L}\text{Cov}(g_{ijl}, g_{ijk})\right); &(222)\\
&= w^2\left[L2f(1-f)(1+m) + 4L(L-1)f(1-f)m\right]; &(223)\\
&= V_0\left[1+m+2(L-1)m\right]; &(224)\\
&= V_0[1+(2L-1)m]; &(225)\\
&= \frac{V_0}{1-r_{am}+r_{am}/2L}; &(226)
\end{aligned}$$

where we have substituted in the equilibrium value of m (Equation 183) and simplified to reach this result.

We therefore have that for large L,

$$\text{Var}(\text{PGI}_{ij}) \approx \frac{V_0}{1-r_{am}}, \tag{227}$$

which agrees with classic results for the inflation of the genetic variance at equilibrium due to assortative mating[5].

A.2 Equilibrium variance of parental PGI

We define $\text{PGI}_{\text{par}(i)} = \text{PGI}_{m(i)} + \text{PGI}_{p(i)}$. Therefore,

$$\text{Var}(\text{PGI}_{\text{par}(i)}) = 2\text{Var}(\text{PGI}_{m(i)}) + 2\text{Cov}(\text{PGI}_{m(i)}, \text{PGI}_{p(i)}). \tag{228}$$

At equilibrium, the variance of the PGI does not change from parents to offspring. Therefore,

$$\text{Var}(\text{PGI}_{\text{par}(i)}) = 2\text{Var}(\text{PGI}_{ij})(1 + r_{am}); \tag{229}$$

$$= 2\frac{1 + r_{am}}{1 - r_{am} + r_{am}/2L}V_0; \tag{230}$$

$$\approx 2\frac{1 + r_{am}}{1 - r_{am}}V_0, \text{ for large } L. \tag{231}$$

A.3 Equilibrium covariance between offspring and parental PGI

Since offspring and parents are symmetrically related,

$$\text{Cov}(\text{PGI}_{ij}, \text{PGI}_{\text{par}(i)}) = 2\text{Cov}(\text{PGI}_{ij}, \text{PGI}_{m(i)}). \tag{232}$$

We can derive that $\text{Corr}(\text{PGI}_{ij}, \text{PGI}_{m(i)}) = \frac{1+r_{am}}{2}$. Therefore,

$$\text{Cov}(\text{PGI}_{ij}, \text{PGI}_{m(i)}) = \frac{1 + r_{am}}{2(1 - r_{am} + r_{am}/2L)}V_0, \tag{233}$$

and therefore

$$\text{Cov}(\text{PGI}_{ij}, \text{PGI}_{\text{par}(i)}) = \frac{1 + r_{am}}{1 - r_{am} + r_{am}/2L}V_0. \tag{234}$$

$$\approx \frac{1 + r_{am}}{1 - r_{am}}V_0, \text{ for large } L. \tag{235}$$

A.4 Equilibrium variance of imputed parental PGI

We now compute the variance of the imputed parental PGI:

$$\text{Var}(\hat{\text{PGI}}_{\text{par}(i)}) = w^2\left[\sum_{l=1}^{L}\text{Var}(\hat{g}_{\text{par}(i)l}) + \sum_{l\neq k}^{L}\text{Cov}(\hat{g}_{\text{par}(i)l}, \hat{g}_{\text{par}(i)k})\right] \tag{236}$$

We compute $\text{Var}(\hat{g}_{\text{par}(i)l})$ separately for each IBD state. If $\text{IBD}_l = 0$, then $\hat{g}_{\text{par}(i)l} = g_{m(i)l} + g_{p(i)l}$, and thus

$$\text{Var}(\hat{g}_{\text{par}(i)l}) = 2\text{Var}(g_{m(i)l}) + 2\text{Cov}(g_{m(i)l}, g_{p(i)l}) \tag{237}$$

$$= 4f(1 - f)(1 + m) + 8mf(1 - f) \tag{238}$$

$$= 4f(1 - f)(1 + 3m). \tag{239}$$

If $\text{IBD}_l = 2$, then $\hat{g}_{\text{par}(i)l} = g_{i1l} + 2f$, and thus $\text{Var}(\hat{g}_{\text{par}(i)l}) = 2f(1 - f)(1 + m)$. If we are in IBD state 1, then $\hat{g}_{\text{par}(i)l} = g_{i1l} + g^k_{i2l} + f$, where g^k_{i2l} is the allele in sibling 2 not IBD with the

alleles in sibling 1. Therefore,

$$\begin{aligned}\text{Var}(\hat{g}_{\text{par}(i)l}) &= \text{Var}(g_{i1l}) + \text{Var}(g_{i2l}^k) + 2\text{Cov}(g_{i1l}, g_{i2l}^k) &(240)\\ &= 2f(1-f)(1+m) + f(1-f) + 4mf(1-f) &(241)\\ &= 3f(1-f)(1+2m). &(242)\end{aligned}$$

Therefore, we have that

$$\text{Var}(\hat{g}_{\text{par}(i)l}) = \begin{cases} 4f(1-f)(1+3m), & \text{if } \text{IBD}_l = 0 \\ 3f(1-f)(1+2m), & \text{if } \text{IBD}_l = 1 \\ 2f(1-f)(1+m). & \text{if } \text{IBD}_l = 2. \end{cases} \tag{243}$$

Since the expectation of $\hat{g}_{\text{par}(i)l}$ does not depend upon the IBD state, we have that

$$\begin{aligned}\text{Var}(\hat{g}_{\text{par}(i)l}) &= f(1-f)[\mathbb{P}(\text{IBD}_l = 0)4(1+3m) + \mathbb{P}(\text{IBD}_l = 1)3(1+2m) + \mathbb{P}(\text{IBD}_l = 2)2(1+m)] &(244)\\ &= 3f(1-f)[1+(13/6)m]. &(245)\end{aligned}$$

We now consider the covariance for distinct loci $l \neq k$. Since the loci segregate independently, $\mathbb{P}(\text{IBD}_l = x, \text{IBD}_k = y) = \mathbb{P}(\text{IBD}_l = x)\mathbb{P}(\text{IBD}_k = y)$. We can therefore compute the covariance by computing the covariance conditional on $\text{IBD}_l = x, \text{IBD}_k = y$ for $x, y \in \{0, 1, 2\}$. Note that, if $\text{IBD}_l = x$ then $\hat{g}_{\text{par}(i)l}$ is comprised of $4 - x$ sibling alleles. Therefore,

$$\text{Cov}(\hat{g}_{\text{par}(i)l}, \hat{g}_{\text{par}(i)k} | \text{IBD}_l = x, \text{IBD}_k = y) = (4-x)(4-y)mf(1-f), \tag{246}$$

and therefore

$$\begin{aligned}\text{Cov}(\hat{g}_{\text{par}(i)l}, \hat{g}_{\text{par}(i)k}) &= mf(1-f)\sum_{x=0}^{2}\sum_{y=0}^{2}(4-x)(4-y)\mathbb{P}(\text{IBD}_l = x)\mathbb{P}(\text{IBD}_k = y) &(247)\\ &= mf(1-f)\sum_{x=0}^{2}(4-x)\mathbb{P}(\text{IBD}_l = x)\sum_{y=0}^{2}(4-y)\mathbb{P}(\text{IBD}_k = y) &(248)\\ &= 9mf(1-f), &(249)\end{aligned}$$

since $\sum_{x=0}^{2}(4-x)\mathbb{P}(\text{IBD}_l = x) = 3$. Therefore, we have that

$$\begin{aligned}\text{Var}(\hat{\text{PGI}}_{\text{par}(i)}) &= w^2\left[L3f(1-f)(1+(13/6)m) + L(L-1)9mf(1-f)\right] &(250)\\ &= w^2 3Lf(1-f)[1+(13/6)m + (L-1)3m] &(251)\\ &= \frac{3}{2}V_0[1+(3L-5/6)m] &(252)\\ &= \frac{3}{2}\frac{1+r_{am}/2 - r_{am}/(12L)}{1-r_{am}+r_{am}/(2L)}V_0 &(253)\\ &\approx \frac{3}{2}\frac{1+r_{am}/2}{1-r_{am}}V_0, \text{ for large } L. &(254)\end{aligned}$$

A.5 Equilibrium covariance between offspring and imputed parental PGI

We now compute

$$\mathrm{Cov}(\mathrm{PGI}_{i1}, \hat{\mathrm{PGI}}_{\mathrm{par}(i)}) = w^2 \left[\sum_{l=1}^{L} \mathrm{Cov}(g_{i1l}, \hat{g}_{\mathrm{par}(i)l}) + 2 \sum_{l=1}^{L} \sum_{k=l+1}^{L} \mathrm{Cov}(g_{i1l}, \hat{g}_{\mathrm{par}(i)k}) \right], \tag{255}$$

since $\mathrm{Cov}(g_{i1l}, \hat{g}_{\mathrm{par}(i)k}) = \mathrm{Cov}(g_{i1k}, \hat{g}_{\mathrm{par}(i)l})$ because we are at equilibrium and all alleles have equal frequency and all loci segregate independently. Following an argument similar to used above, we have that

$$\mathrm{Cov}(g_{i1l}, \hat{g}_{\mathrm{par}(i)l}) = \begin{cases} 2f(1-f)(1+m), & \text{if } \mathrm{IBD}_l = 2 \\ 2f(1-f)(1+2m), & \text{if } \mathrm{IBD}_l = 1 \\ 2f(1-f)(1+3m), & \text{if } \mathrm{IBD}_l = 0. \end{cases} \tag{256}$$

Therefore, $\mathrm{Cov}(g_{i1l}, \hat{g}_{\mathrm{par}(i)l}) = 2f(1-f)(1+2m)$. For $k \neq l$, we have

$$\mathrm{Cov}(g_{i1l}, \hat{g}_{\mathrm{par}(i)k}) = \begin{cases} 4f(1-f)m, & \text{if } \mathrm{IBD}_l = 2 \\ 6f(1-f)m, & \text{if } \mathrm{IBD}_l = 1 \\ 8f(1-f)m, & \text{if } \mathrm{IBD}_l = 0. \end{cases} \tag{257}$$

Therefore, $\mathrm{Cov}(g_{i1l}, \hat{g}_{\mathrm{par}(i)k}) = 6mf(1-f)$. We therefore have that

$$\begin{aligned} \mathrm{Cov}(\mathrm{PGI}_{i1}, \hat{\mathrm{PGI}}_{\mathrm{par}(i)}) &= w^2 \left[2Lf(1-f)(1+2m) + 6L(L-1)mf(1-f) \right]; && (258) \\ &= V_0[1 + 2m + 3(L-1)m]; && (259) \\ &= V_0[1 + (3L-1)m]; && (260) \\ &= \frac{1 + r_{am}/2}{1 - r_{am} + r_{am}/(2L)} V_0; && (261) \\ &\approx \frac{1 + r_{am}/2}{1 - r_{am}} V_0, \text{ for large } L. && (262) \end{aligned}$$

A.6 Equilibrium covariance between imputed and observed parental PGI

We now compute

$$\mathrm{Cov}(\mathrm{PGI}_{\mathrm{par}(i)}, \hat{\mathrm{PGI}}_{\mathrm{par}(i)}) = w^2 \left[\sum_{l=1}^{L} \mathrm{Cov}(g_{\mathrm{par}(i)l}, \hat{g}_{\mathrm{par}(i)l}) + 2 \sum_{l=1}^{L} \sum_{k=l+1}^{L} \mathrm{Cov}(g_{\mathrm{par}(i)l}, \hat{g}_{\mathrm{par}(i)k}) \right], \tag{263}$$

since $\text{Cov}(g_{\text{par}(i)l}, \hat{g}_{\text{par}(i)k}) = \text{Cov}(g_{\text{par}(i)k}, \hat{g}_{\text{par}(i)l})$ because we are at equilibrium and all alleles have equal frequency and all loci segregate independently. Following an argument similar to used above, we have that

$$\text{Cov}(g_{\text{par}(i)l}, \hat{g}_{\text{par}(i)l}) = \begin{cases} 2f(1-f)(1+3m), & \text{if IBD}_l = 2 \\ 3f(1-f)(1+3m), & \text{if IBD}_l = 1 \\ 4f(1-f)(1+3m), & \text{if IBD}_l = 0. \end{cases} \tag{264}$$

Therefore, we have that $\text{Cov}(g_{\text{par}(i)l}, \hat{g}_{\text{par}(i)l}) = 3f(1-f)(1+3m)$. For $l \neq k$, we have that

$$\text{Cov}(g_{\text{par}(i)l}, \hat{g}_{\text{par}(i)k}) = \begin{cases} 16f(1-f)m, & \text{if IBD}_l = 0 \\ 12f(1-f)m, & \text{if IBD}_l = 1 \\ 8f(1-f)m, & \text{if IBD}_l = 2. \end{cases} \tag{265}$$

Therefore, we have that $\text{Cov}(g_{\text{par}(i)l}, \hat{g}_{\text{par}(i)k}) = 12f(1-f)m$. This gives

$$\begin{aligned} \text{Cov}(\text{PGI}_{\text{par}(i)}, \hat{\text{PGI}}_{\text{par}(i)}) &= w^2\left[L3f(1-f)(1+3m) + 12L(L-1)f(1-f)m\right]; && (266)\\ &= \frac{3}{2}V_0[1+3m+4(L-1)m]; && (267)\\ &= \frac{3}{2}V_0[1+(4L-1)m]; && (268)\\ &= \frac{3}{2}\frac{1+r_{am}}{1-r_{am}+(r_{am}/2L)}V_0; && (269)\\ &\approx \frac{3}{2}\frac{1+r_{am}}{1-r_{am}}V_0, \text{ for large } L. && (270) \end{aligned}$$

B Imputation from one parent and multiple offspring

Here we give the probabilities and expectations of the missing father's genotype conditional on observing the mother and two sibling offspring. Here, we do not assume we can infer which allele is shared when pairs are IBD1 and both heterozygous, so these tables are appropriate for imputation with un-phased data. We generated the tables using an automated application of Bayes' Rule.

B.1 With IBD = 0

$$g_{p(i)} + g_{m(i)} = (g_{i1} + g_{i2}) \Rightarrow g_{p(i)} = (g_{i1} + g_{i2}) - g_{m(i)} \tag{271}$$

B.2 With IBD = 1

$\{g_{p(i)}, g_{m(i)}\}$	g_{i1}, g_{i2}	$P(g_{i1}, g_{i2} \vert g_{m(i)}, g_{p(i)}, IBD = 1)$
$[0, 0]$	$[0, 0]$	1
$[0, 1]$	$[0, 0]$	0.25
	$[0, 1]$	0.5
	$[1, 1]$	0.25
$[0, 2]$	$[1, 1]$	1
$[1, 1]$	$[0, 1]$	0.5
	$[1, 2]$	0.5
$[1, 2]$	$[1, 1]$	0.25
	$[1, 2]$	0.5
	$[2, 2]$	0.25
$[2, 2]$	$[2, 2]$	1

$g_{p(i)}$	$g_{m(i)}$	g_{i1}, g_{i2}	$P(g_{p(i)} \| g_{m(i)}, g_{i1}, g_{i2}, IBD = 1)$
0	0	[0, 0]	$\frac{1*(1-f)^2}{1*(1-f)^2+0.25*2f(1-f)}$
1	0	[0, 0]	$\frac{0.25*2f(1-f)}{1*(1-f)^2+0.25*2f(1-f)}$
2	0	[0, 0]	0
0	0	[1, 0]	0
1	0	[1, 0]	1
2	0	[1, 0]	0
0	0	[0, 2]	0
1	0	[0, 2]	0
2	0	[0, 2]	0
0	0	[1, 2]	0
1	0	[1, 2]	0
2	0	[1, 2]	0
0	0	[2, 2]	0
1	0	[2, 2]	0
2	0	[2, 2]	0
0	1	[0, 0]	1
1	1	[0, 0]	0
2	1	[0, 0]	0
0	1	[1, 0]	$\frac{0.5*(1-f)^2}{0.5*(1-f)^2+0.5*2f(1-f)}$
1	1	[1, 0]	$\frac{0.5*2f(1-f)}{0.5*(1-f)^2+0.5*2f(1-f)}$
2	1	[1, 0]	0
0	1	[0, 2]	0
1	1	[0, 2]	0
2	1	[0, 2]	0
0	1	[1, 2]	0
1	1	[1, 2]	$\frac{0.5*2f(1-f)}{0.5*2f(1-f)+0.5*f^2}$
2	1	[1, 2]	$\frac{0.5*f^2}{0.5*2f(1-f)+0.5*f^2}$
0	1	[2, 2]	0
1	1	[2, 2]	0
2	1	[2, 2]	1
0	2	[0, 0]	0
1	2	[0, 0]	0
2	2	[0, 0]	0
0	2	[1, 0]	0
1	2	[1, 0]	0
2	2	[1, 0]	0
0	2	[0, 2]	0
1	2	[0, 2]	0
2	2	[0, 2]	0
0	2	[1, 2]	0
1	2	[1, 2]	1
2	2	[1, 2]	0
0	2	[2, 2]	0
1	2	[2, 2]	$\frac{0.25*2f(1-f)}{0.25*2f(1-f)+1*f^2}$
2	2	[2, 2]	$\frac{1*f^2}{0.25*2f(1-f)+1*f^2}$

$g_{m(i)}$	g_{i1}, g_{i2}	$E[g_{p(i)} \vert g_{m(i)}, g_{i1}, g_{i2}]$
0	$[0, 2]$	0
0	$[0]$	$\frac{0.25*2f(1-f)}{1*(1-f)^2+0.25*2f(1-f)} * 1$
0	$[1, 0]$	$1 * 1$
0	$[1, 2]$	0
0	$[1]$	$\frac{0.25*2f(1-f)}{0.25*2f(1-f)+1*f^2} * 1 + \frac{1*f^2}{0.25*2f(1-f)+1*f^2} * 2$
0	$[2]$	0
1	$[0, 2]$	0
1	$[0]$	0
1	$[1, 0]$	$\frac{0.5*2f(1-f)}{0.5*(1-f)^2+0.5*2f(1-f)} * 1$
1	$[1, 2]$	$\frac{0.5*2f(1-f)}{0.5*2f(1-f)+0.5*f^2} * 1 + \frac{0.5*f^2}{0.5*2f(1-f)+0.5*f^2} * 2$
1	$[1]$	$\frac{0.25*f^2}{0.25*(1-f)^2+0.25*f^2} * 2$
1	$[2]$	$1 * 2$
2	$[0, 2]$	0
2	$[0]$	0
2	$[1, 0]$	0
2	$[1, 2]$	$1 * 1$
2	$[1]$	$\frac{0.25*2f(1-f)}{1*(1-f)^2+0.25*2f(1-f)} * 1$
2	$[2]$	$\frac{0.25*2f(1-f)}{0.25*2f(1-f)+1*f^2} * 1 + \frac{1*f^2}{0.25*2f(1-f)+1*f^2} * 2$

B.3 With IBD = 2

$\{g_{m(i)}, g_{p(i)}\}$	g_{i1}, g_{i2}	$P(g_{i1}, g_{i2} \vert g_{m(i)}, g_{p(i)}, IBD = 2)$
[0,0]	0	1
[0,1]	0	0.5
[0,1]	1	0.5
[0,2]	1	1
[1,1]	0	0.25
[1,1]	1	0.5
[1,1]	2	0.25
[1,2]	1	0.5
[1,2]	2	0.5
[2,2]	2	1

$g_{p(i)}$	$g_{m(i)}$	g_{i1}, g_{i2}	$P(g_{p(i)} \mid g_{m(i)}, g_{i1}, g_{i2}, IBD = 2)$
0	0	0	$\frac{1*(1-f)^2}{1*(1-f)^2+0.5*2f(1-f)}$
1	0	0	$\frac{0.5*2f(1-f)}{1*(1-f)^2+0.5*2f(1-f)}$
2	0	0	0
0	0	1	0
1	0	1	$\frac{0.5*2f(1-f)}{0.5*2f(1-f)+1*f^2}$
2	0	1	$\frac{1*f^2}{0.5*2f(1-f)+1*f^2}$
0	0	2	0
1	0	2	0
2	0	2	0
0	1	0	$\frac{0.5*(1-f)^2}{0.5*(1-f)^2+0.25*2f(1-f)}$
1	1	0	$\frac{0.25*2f(1-f)}{0.5*(1-f)^2+0.25*2f(1-f)}$
2	1	0	0
0	1	1	$\frac{0.5*(1-f)^2}{0.5*(1-f)^2+0.5*2f(1-f)+0.5*f^2}$
1	1	1	$\frac{0.5*2f(1-f)}{0.5*(1-f)^2+0.5*2f(1-f)+0.5*f^2}$
2	1	1	$\frac{0.5*f^2}{0.5*(1-f)^2+0.5*2f(1-f)+0.5*f^2}$
0	1	2	0
1	1	2	$\frac{0.25*2f(1-f)}{0.25*2f(1-f)+0.5*f^2}$
2	1	2	$\frac{0.5*f^2}{0.25*2f(1-f)+0.5*f^2}$
0	2	0	0
1	2	0	0
2	2	0	0
0	2	1	$\frac{1*(1-f)^2}{1*(1-f)^2+0.5*2f(1-f)}$
1	2	1	$\frac{0.5*2f(1-f)}{1*(1-f)^2+0.5*2f(1-f)}$
2	2	1	0
0	2	2	0
1	2	2	$\frac{0.5*2f(1-f)}{0.5*2f(1-f)+1*f^2}$
2	2	2	$\frac{1*f^2}{0.5*2f(1-f)+1*f^2}$

$g_{m(i)}$	g_{i1}, g_{i2}	$E[g_{p(i)} \vert g_{m(i)}, g_{i1}, g_{i2}, IBD=2]$
0	0	$\frac{0.5*2f(1-f)}{1*(1-f)^2+0.5*2f(1-f)} * 1$
0	1	$\frac{0.5*2f(1-f)}{0.5*2f(1-f)+1*f^2} * 1 + \frac{1*f^2}{0.5*2f(1-f)+1*f^2} * 2$
0	2	0
1	0	$\frac{0.25*2f(1-f)}{0.5*(1-f)^2+0.25*2f(1-f)} * 1$
1	1	$\frac{0.5*2f(1-f)}{0.5*(1-f)^2+0.5*2f(1-f)+0.5*f^2} * 1 + \frac{0.5*f^2}{0.5*(1-f)^2+0.5*2f(1-f)+0.5*f^2} * 2$
1	2	$\frac{0.25*2f(1-f)}{0.25*2f(1-f)+0.5*f^2} * 1 + \frac{0.5*f^2}{0.25*2f(1-f)+0.5*f^2} * 2$
2	0	0
2	1	$\frac{0.5*2f(1-f)}{1*(1-f)^2+0.5*2f(1-f)} * 1$
2	2	$\frac{0.5*2f(1-f)}{0.5*2f(1-f)+1*f^2} * 1 + \frac{1*f^2}{0.5*2f(1-f)+1*f^2} * 2$

C Imputation without IBD

If we find a genotype-IBD state that is impossible given the laws of Mendelian inheritance (for example, two siblings with genotypes 0 and 2 and IBD state 2) we assume that there is an error in the IBD inference, and we proceed to impute the missing parental genotypes without using IBD information.

For imputation of parents from a set of siblings, we compute the probabilities of the parents' genotypes given the siblings' genotypes (g_{i1}, g_{i2},...):

$$P(g_{p(i)}, g_{m(i)} | g_{i1}, g_{i2}, ...) = \frac{P(g_{i1}, g_{i2}, ... | g_{p(i)}, g_{m(i)})}{P(g_{i1}, g_{i2}, ...)} P(g_{p(i)}, g_{m(i)}) \tag{272}$$

$$= \frac{\prod_j P(g_{ij} | g_{p(i)}, g_{m(i)}) P(g_{p(i)}, g_{m(i)})}{\sum_{g_{p(i)}} \sum_{g_{m(i)}} \prod_j P(g_{ij} | g_{p(i)}, g_{m(i)}) P(g_{p(i)}, g_{m(i)})}. \tag{273}$$

For imputation of a parent (taken to be the father here) given the genotypes of the mother and the offspring, we compute the probabilities of the missing father's genotype:

$$P(g_{p(i)} | g_{m(i)}, g_{i1}, g_{i2}, ...) = \frac{P(g_{p(i)}, g_{m(i)}) \prod_{g_{ij}} P(g_{ij} | g_{p(i)}, g_{m(i)})}{\sum_{g_{p(i)}} P(g_{p(i)}, g_{m(i)}) \prod_{g_{ij}} P(g_{ij} | g_{p(i)}, g_{m(i)})} \tag{274}$$

Assuming random-mating, $P(g_{p(i)}, g_{m(i)}) = \binom{2}{g_{p(i)}} f^{g_{p(i)}} (1-f)^{2-g_{p(i)}} \binom{2}{g_{m(i)}} f^{g_{m(i)}} (1-f)^{2-g_{m(i)}}$. The only remaining term to compute is $P(g_{ij} | g_{p(i)}, g_{m(i)})$, which can be computed using Mendelian Laws of Inheritance. The imputed values are then the expectations of the parental genotypes over these conditional probability distributions.

D Linear Imputation

Unbiased linear imputations of parental genotypes can be derived from the variance-covariance matrix of the sibling and parental genotypes:

$$\text{Var}\left(X_i = \begin{bmatrix} g_{i1} \\ g_{i2} \\ g_{p(i)} \\ g_{m(i)} \end{bmatrix}\right) = 2f(1-f)\begin{bmatrix} 1 & 0.5 & 0.5 & 0.5 \\ 0.5 & 1 & 0.5 & 0.5 \\ 0.5 & 0.5 & 1 & 0 \\ 0.5 & 0.5 & 0 & 1 \end{bmatrix}. \tag{275}$$

If we partition the variables into two groups, X_{i1} and X_{i2}, with the covariance matrix partitioned into

$$\text{Var}\left(\begin{bmatrix} X_{i1} \\ X_{i2} \end{bmatrix}\right) = \begin{bmatrix} \Sigma_{11} & \Sigma_{12} \\ \Sigma_{12}^T & \Sigma_{22} \end{bmatrix} \tag{276}$$

then we can apply the formula for $\mathbb{E}[X_2|X_1]$ as if they are jointly multivariate Gaussian random variables to derive an unbiased imputation of X_2 that is a linear function of X_1:

$$\mathbb{E}[X_2|X_1] = \mathbb{E}[X_2] + \Sigma_{12}^T\Sigma_{11}^{-1}(X_1 - \mathbb{E}[X_1]). \tag{277}$$

D.1 Imputation from parent-offspring pairs

Consider imputing $g_{p(i)}$ by a linear function of g_{i1} and $g_{m(i)}$:

$$\hat{g}_{p(i)} = \frac{4f + 2g_{i1} - g_{m(i)}}{3}, \tag{278}$$

which has variance $\frac{2}{3}f(1-f)$, which is 1/3 of the variance of $g_{p(i)}$.

D.2 Imputation from sibling pairs

For $g_{\text{par}(i)} = g_{p(i)} + g_{m(i)}$, we have that

$$\text{Var}\left(\begin{bmatrix} g_{i1} \\ g_{i2} \\ g_{\text{par}(i)} \end{bmatrix}\right) = 2f(1-f)\begin{bmatrix} 1 & 0.5 & 1 \\ 0.5 & 1 & 1 \\ 1 & 1 & 2 \end{bmatrix}. \tag{279}$$

From this, we impute $g_{\text{par}(i)}$ as a linear function of g_{i1} and g_{i2}:

$$\hat{g}_{\text{par}(i)} = \frac{4f + 2(g_{i1} + g_{i2})}{3}, \tag{280}$$

which has variance $\frac{8}{3}f(1-f)$, which is 2/3 of the variance of $g_{\text{par}(i)}$.
