# Supplementary material for: Mendelian imputation of parental genotypes improves estimates of direct genetic effects
Source: Nat Genet. 2022 Jun 9;54(6):897–905. doi: 10.1038/s41588-022-01085-0 (PMC9197765; doi:10.1038/s41588-022-01085-0)
Supplement: Supplementary file 2 — Reporting Summary [file 41588_2022_1085_MOESM2_ESM.pdf]

nature research

Corresponding author(s): Alexander I. Young, Augustine Kong

Last updated by author(s): Mar 29, 2022

Reporting Summary

Nature Research wishes to improve the reproducibility of the work that we publish. This form provides structure for consistency and transparency in reporting. For further information on Nature Research policies, see our Editorial Policies and the Editorial Policy Checklist.

Statistics

For all statistical analyses, confirm that the following items are present in the figure legend, table legend, main text, or Methods section.

n/a	Confirmed	
☐	☒	The exact sample size (*n*) for each experimental group/condition, given as a discrete number and unit of measurement
☒	☐	A statement on whether measurements were taken from distinct samples or whether the same sample was measured repeatedly
☐	☒	The statistical test(s) used AND whether they are one- or two-sided<br>*Only common tests should be described solely by name; describe more complex techniques in the Methods section.*
☐	☒	A description of all covariates tested
☐	☒	A description of any assumptions or corrections, such as tests of normality and adjustment for multiple comparisons
☐	☒	A full description of the statistical parameters including central tendency (e.g. means) or other basic estimates (e.g. regression coefficient) AND variation (e.g. standard deviation) or associated estimates of uncertainty (e.g. confidence intervals)
☐	☒	For null hypothesis testing, the test statistic (e.g. *F*, *t*, *r*) with confidence intervals, effect sizes, degrees of freedom and *P* value noted<br>*Give P values as exact values whenever suitable.*
☒	☐	For Bayesian analysis, information on the choice of priors and Markov chain Monte Carlo settings
☒	☐	For hierarchical and complex designs, identification of the appropriate level for tests and full reporting of outcomes
☐	☒	Estimates of effect sizes (e.g. Cohen's *d*, Pearson's *r*), indicating how they were calculated

Our web collection on statistics for biologists contains articles on many of the points above.

Software and code

Policy information about availability of computer code

Data collection	No new data was collected in this study, so no software was used for data collection.
Data analysis	We used snipar v0.0.9 (https://doi.org/10.5281/zenodo.6484858) for IBD inference, imputation, genome-wide association and polygenic index analyses, and estimation of genome-wide correlations between effects. snipar is available as a Python package under an MIT license at https://github.com/AlexTISYoung/snipar. Analyses were performed using Anaconda3 with Python 3.7.6 (https://repo.anaconda.com/archive/). Phasing was performed using SHAPEIT v2.r904 (https://mathgen.stats.ox.ac.uk/genetics_software/shapeit/shapeit.html), and haplotype merging was performed using QCTOOL v2.0.7 (https://www.well.ox.ac.uk/~gav/qctool_v2/). Relationship inference was performed using KING 2.2.4 (http://people.virginia.edu/~wc9c/KING). LD scores were computed using LDSC v1.0 (https://github.com/bulik/ldsc). Genome-wide association analyses in the sample of unrelated individuals was performed using BOLT v2.3.4 (https://alkesgroup.broadinstitute.org/BOLT-LMM/).

For manuscripts utilizing custom algorithms or software that are central to the research but not yet described in published literature, software must be made available to editors and reviewers. We strongly encourage code deposition in a community repository (e.g. GitHub). See the Nature Research guidelines for submitting code & software for further information.

Data

Policy information about availability of data

All manuscripts must include a data availability statement. This statement should provide the following information, where applicable:
- Accession codes, unique identifiers, or web links for publicly available datasets
- A list of figures that have associated raw data
- A description of any restrictions on data availability

Summary statistics for the direct effects, NTCs, and population effects of 1,586,010 SNPs on 9 phenotypes can be downloaded from http://www.thessgac.org/data,

subject to a terms of use to encourage responsible use of the data. Applications for access to the UK Biobank data can be made on the UK Biobank website: http://www.ukbiobank.ac.uk/register-apply/.

Field-specific reporting

Please select the one below that is the best fit for your research. If you are not sure, read the appropriate sections before making your selection.

☒ Life sciences ☐ Behavioural & social sciences ☐ Ecological, evolutionary & environmental sciences

For a reference copy of the document with all sections, see nature.com/documents/nr-reporting-summary-flat.pdf

Life sciences study design

All studies must disclose on these points even when the disclosure is negative.

Sample size	We used all genotyped individuals in UK Biobank passing ancestry and quality filters with at least one genotyped first-degree relative: 39,619.
Data exclusions	In order to make our analyses comparable to existing genome-wide association studies, which generally use a genetically homogeneous population, we restricted our sample to individuals determined by UK Biobank to have predominantly White British ancestry. We filtered out individuals identified by UK Biobank as having excess relatives, excess heterozygosity, or sex chromosome aneuploidy.
Replication	Replication is not applicable to this study since we are describing a novel methodology. We demonstrate the methodology with application to UK Biobank data, and the results presented are valid for that dataset. Different results may be obtained by application of this methodology to different datasets/populations.
Randomization	Genetic material is randomized during meiosis, which we use to obtain estimates of direct genetic effects, i.e. to remove the influence of confounding factors.
Blinding	Blinding is not applicable since we did not compare different experimental groups.

Reporting for specific materials, systems and methods

We require information from authors about some types of materials, experimental systems and methods used in many studies. Here, indicate whether each material, system or method listed is relevant to your study. If you are not sure if a list item applies to your research, read the appropriate section before selecting a response.

Materials & experimental systems

n/a	Involved in the study	
☒	☐	Antibodies
☒	☐	Eukaryotic cell lines
☒	☐	Palaeontology and archaeology
☒	☐	Animals and other organisms
☒	☐	Human research participants
☒	☐	Clinical data
☒	☐	Dual use research of concern

Methods

n/a	Involved in the study	
☒	☐	ChIP-seq
☒	☐	Flow cytometry
☒	☐	MRI-based neuroimaging
